# A 1.2-V 7.76-ENOB 1-MS/s single-ended SAR ADC in 65-nm CMOS for biomedical applications

**DOI:** 10.1038/s41598-025-21817-6

**Published:** 2025-11-05

**Authors:** Kawther I. Arafa, Dina M. Ellaithy, Heba Shawkey, Mohamed Abouelatta, Abdelhalim Zekry

**Affiliations:** 1 Electronics and Communications Department, Faculty of Engineering, Ain Shams University, Cairo, Egypt; 2 Microelectronics Department, Electronics Research Institute (ERI), Cairo, 11843 Egypt; 3 Faculty of Engineering, Egypt University of Informatics, Cairo, Egypt

**Keywords:** Successive approximation register analog-to-digital converter (SAR ADC), Bootstrap sample-and-hold switch (S/H), Dynamic comparator, Capacitive digital-to-analog converter (CDAC), Low power, Area, Biomedical engineering, Electrical and electronic engineering

## Abstract

A successive approximation register analog-to-digital converter (SAR ADC) is a promising approach used in biomedical applications due to its energy-efficiency architecture with less complex hardware implementation. The core building blocks of SAR ADC are sample-and-hold switch (S/H), comparator, logic control register, and digital-to-analog converter (DAC). To enhance the overall performance, a high-isolation CMOS bootstrap S/H switch has been used. The SFDR of proposed ADC has increased by up to 2.2 dB. Also, we propose a double-tail single-ended dynamic latch comparator with extra pair PMOS transistors that save power by up to 7.5% as compared to the traditional double-tail dynamic comparator. Moreover, after adding these pair of transistors into conventional double tail dynamic comparator without any calibration cost, the SNDR has increased by more than 2 dB and 0.3bit improvement of ENOB. Furthermore, a synchronous modified SAR logic control register based on low-power D flip-flops (FFs) is proposed. A metal-isolator-metal capacitor (MIM) with a modified capacitance reduction configuration is used to improve the active area of the capacitive DAC (CDAC) compared to the conventional CDAC with 36.7% saving power. The proposed ADC has been implemented using a 65-nm TSMC CMOS process, 1.2 V supply voltage with a sampling rate of 1 MS/s. An active area of 0.00585 mm^2^ with a total post-result power consumption of 5.75 µW has been accomplished for the proposed fully integrated ADC.

## Introduction

**Fig. 1:**
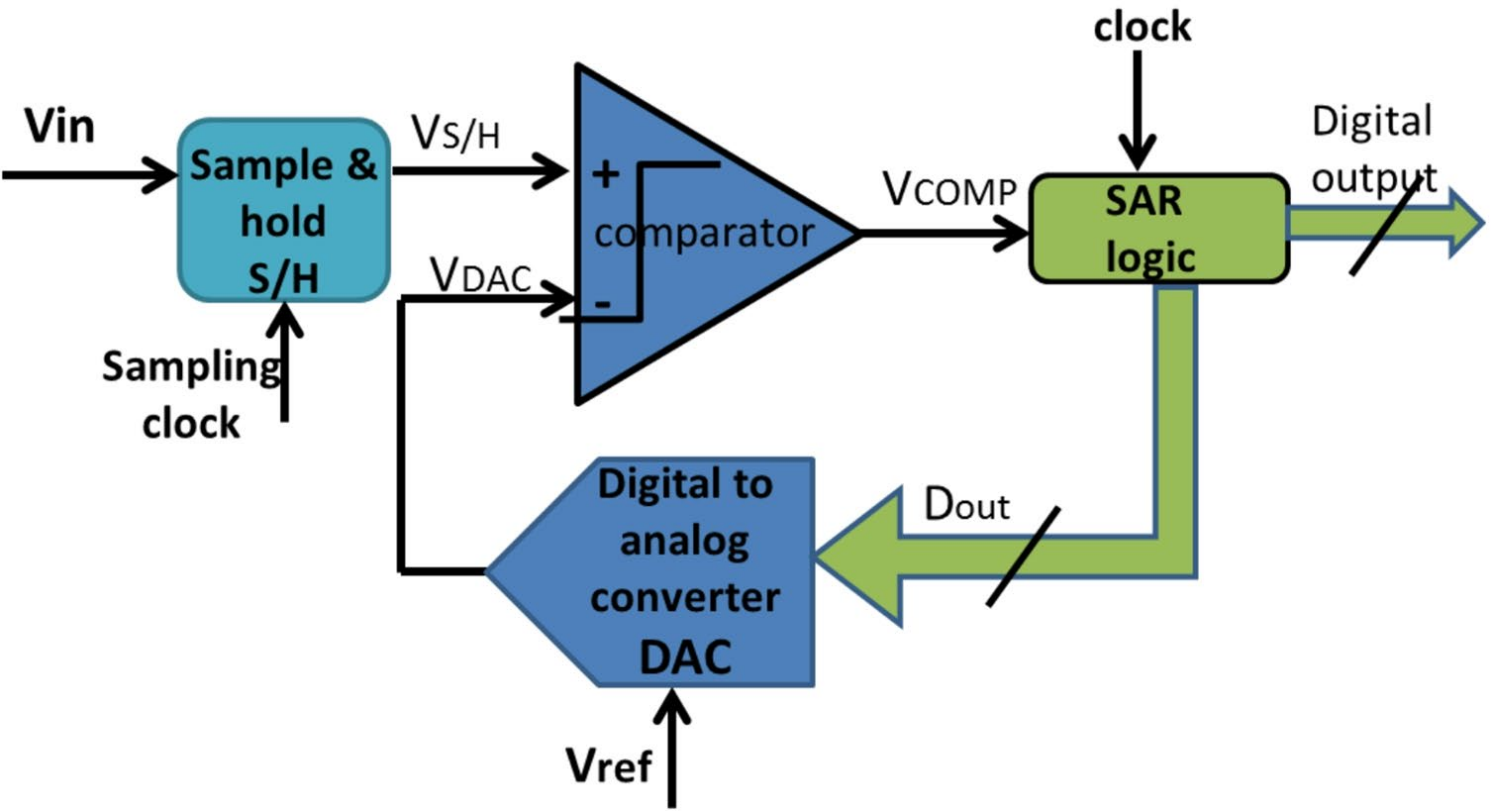
Architecture of SAR ADC^1^.

 The energy efficiency of the successive approximation register analog-to-digital converter SAR ADC results in the wide employment of this type of ADC in biomedical applications. The straightforward implementation of SAR ADC reduces power and area overhead compared to different ADC architectures^1^. For the optimum exploitation of SAR ADC, the resolution should be limited. As shown in Fig. 1, the normal operation of SAR ADC is started by a sample-and-hold switch (S/H) to partition the analog input into samples. Each sample has been compared to the output produced from a digital-to-analog converter (DAC) which corresponds to the half of the supply voltage at the initial instant^2^. The comparator has generated a decision indicating if the sample of analog input is greater or less than the output voltage from the DAC. Then, the comparator output has fed to the logic control register that includes several numbers of registers to produce a digital code^3^. The corresponding analog value of this digital code generated by the DAC has been feedback again to one terminal of the comparator input to be compared with the input sample once more^4^. This loop continues repeated until the analog value corresponding to the output digital code goes close to the input sample value.

A great effort has been made to decrease the total power consumption, area, and delay while obtaining a high-performance SAR ADC^1–6^. Previous studies show that the overall differential architecture enhance the ADC performance by reducing the even-order harmonics. Also, it is superior to common-mode noise rejection; however, these advantages come at the cost of power consumption, area, and input capacitance size ($\:{2}^{N}{C}_{u}$)^3^. Therefore, it is not the recommended choice for implantable biosensor systems with limited size and power. In contrast, the single-ended architecture can digitize the input signal with a much smaller input capacitance of ($\:{2}^{N-1\:}{C}_{u}$), resulting in significant power and area savings. So, it has gained a particular attenuation as compared to differential architecture. Recently, several works^2–6^ have proposed an energy-efficient single ended SAR-ADC for biomedical applications.

In addition, various schemes have proposed different S/H implementations to enhance the overall ADC performance^7–12^. Utilizing the CMOS transmission gates can achieve power reduction. However, it limits the resolution of the ADC due to the variation of conductivity depending on the input signal^1,7^. Otherwise, a bootstrap switch is used to reduce this variation and enhance the linearity^12^. The design of the bootstrapped switch has a great impact on the spurious-free-dynamic range (SFDR) of the SAR-ADC^8–12^. As well, a dual bootstrapped S/H switch has been employed to improve linearity and decrease distortion, but it increases the design’s power consumption^8–11^. In the biomedical application, power consumption reduction is the main objective of the design. Consequently, the bootstrap circuit is the typical architecture for the SAR sample and hold switch.

Since the SAR ADC contains two power-consumer sub-blocks, the digital-to-analog converter (DAC) and the comparator, most of previous works have employed energy-efficient implementation schemes for these sub-blocks^2–40^. As the comparator is the only fully analog component in SAR ADCs, to enhance the SAR-ADC’s accuracy and linearity, and consequently improve the SNDR of the SAR-ADC, attention must be paid to various dynamic considerations, including offset voltage, noise, power consumption, and speed in the comparator block^2,5,14–19^. Various research papers have utilized the dynamic latch comparator due to its’ power efficiency compared to traditional comparators^2–35^. The dynamic comparators consume less power than static comparators, which makes them valuable in the biomedical industry. Furthermore, different dynamic latch comparator configurations enhance the performance with respect to kick-back noise, input-referred offset voltage, resolution, and speed^1–5^. A single-stage dynamic comparator has been employed to save both power consumption and area but sacrifice precision^6^. To compromise between power consumption, offset voltage, and noise, a two-stage dynamic comparator is employed, in which the pre-amplifier and dynamic comparator are connected in cascaded^26,35^. In addition, a multi-stage preamplifier dynamic comparator has been employed to further reduce noise and input referred offset voltage, although it increases the power consumption and area^5^.

Replacing the batteries in implanted biomedical sensors, which requires surgery, is costly and dangerous. Therefore, low-power circuit design is essential to realize a long battery lifetime. Hence, several research have been proposed energy efficient DAC for biomedical applications^1,3–5^. It is responsible for converting the applied digital bits into an analog output voltage by the aid of an array of capacitors or resistors and a reference voltage. Actually, for simple and fast conversion, a resistor-based DAC is employed. Nevertheless, the negative aspects include high power consumption, wide area, low stability, nonlinearity, and matching requirements^1^. In order to have significant power and area savings, a capacitor array in place of the resistance array has been used^11^. The binary-weighted capacitive DAC (CDAC) with different configurations are proposed to reduce energy consumption^2–5^. Moreover, a two-stage subarray capacitor may be used to mitigate the parasitic capacitor effect and enhance linearity^6^. Also, using a bridge capacitor may decrease the die area and boost energy savings^3,14,22^. For high resolution, the dual-split CDAC array capacitor is employed to minimize the area^11,25^. Moreover, the memristor DAC technique has been utilized to decrease area beyond the parasitic capacitor effect^20^. Likewise, the switching scheme significantly affects the performance of the DAC. In the two-level switching scheme CDAC reported in^25^, when a trial capacitor switching is reversed and another attempt at switching is performed with the following capacitor, excessive power consumption results and high stability and accuracy of CDAC compared to other level schemes. So, to reduce the switching energy and overall area of CDAC, three-level^5,17^ and four-level^21,22^ are implemented. However, the linearity of SAR-ADC may be limited by the reference voltage’s accuracy. Various efforts have been made to decrease the energy of the capacitor array and enhance switching performance^5,17,21,22,25^.

According to the implementation of SAR logic, SAR ADC operates in the synchronous mechanism or in the asynchronous mechanism. For the simplicity of design, the synchronous SAR ADC was employed to avoid metastability and good linearity. Various research works have proposed different synchronous architectures of the SAR logic to save power^10,16^. On the other hand, asynchronous SAR ADC is widely used to accomplish faster bit conversion with an optimized time sequence^5,11,12,17^. The fast speed of SAR ADC is achieved by asynchronous SAR logic at a susceptibility to exposed noise and jitter. Thus, the suitable implementation of the SAR logic for biomedical applications is the synchronous mechanism.

In this paper, a low-power single-ended SAR ADC in 65-nm CMOS for biomedical applications is presented. A high-isolation bootstrapped S/H switch is proposed to separate the gate of the sampled switch from any disturbance and maintain stationary during the holding phase. In addition, the overall linearity of the proposed ADC is enhanced. The dynamic latch comparator is implemented with a modified double-tail architecture to save power, time, and decrease the kickback noise as compared to the conventional dynamic latch comparator. Furthermore, a synchronous successive approximation register based on a low-power D-flip flop is proposed. Proceeding with the same strategy of limiting power consumption and diminishing area with good performance, the capacitive digital-to-analog converter (CDAC) with a high-speed switching mechanism is employed in the proposed SAR ADC.

The rest of this paper is organized as follows. The following section demonstrates the design of the proposed SAR ADC with a detailed explanation of the structure of each key component. The high-isolation bootstrapped S/H switch, the double-tail with extra pair PMOS transistors dynamic comparator, the high-speed CDAC, and the low-power SAR control logic have been implemented using a 65-nm CMOS process with 1.2-V supply voltage and presented in “Post-Simulation Results and Comparisons”. Besides the simulation results of the low-power 8-bit single-ended SAR ADC, a performance comparison of the proposed ADC with the state-of-the-art is listed in this section. In “Conclusion”, this section concludes the paper.

## Architecture of proposed SAR ADC

We propose a low-power single-ended 8-bit SAR ADC appropriate for biomedical applications. In this section the proposed architecture of key blocks of SAR ADC is presented.

### High-isolation CMOS bootstrap S/H switch

The sample and hold block (S/H) is considered the essential bottleneck in the SAR ADC, which dominates the accuracy and resolution of the ADC. The conventional bootstrapped S/H switch is widely used for being extremely energy efficient with low-medium resolution and improved speed which is suitable for biomedical applications^2^. The straightforward concept of this sampler is that the variations of the on-resistance of the sampling switch must be kept constant to ensure low distortion and high linearity in the system^7^. The on-resistor (R_on_) of the sampling MOS switch depends on the voltage between the gate and source (V_GS_), which is presented in Eq. (1)^7^:

1 $$\:{R}_{on}\:=\:{\left({\mu\:}_{n}{C}_{ox}\frac{W}{L}\right({V}_{GS}-{V}_{th}\left)\right)}^{-1}$$

Where $\:\varvec{\mu\:}$ is the surface mobility, $\:{\varvec{C}}_{\varvec{o}\varvec{x}}$ is the capacitance per unit gate area and $\:{\varvec{V}}_{th}$ the threshold voltage of MOSFET. As well, the charge injection depends on the variations of the gate and source voltage (V_GS_).

Also, any variation of the input signal should be avoided to diminish the charge injection errors^8^. Equation (2) presents the charge injection of a sample switch^8^:

2 $$\:{Q}_{I}=\:{C}_{ox}WL({V}_{GS}-{V}_{th})$$

**Fig. 2:**
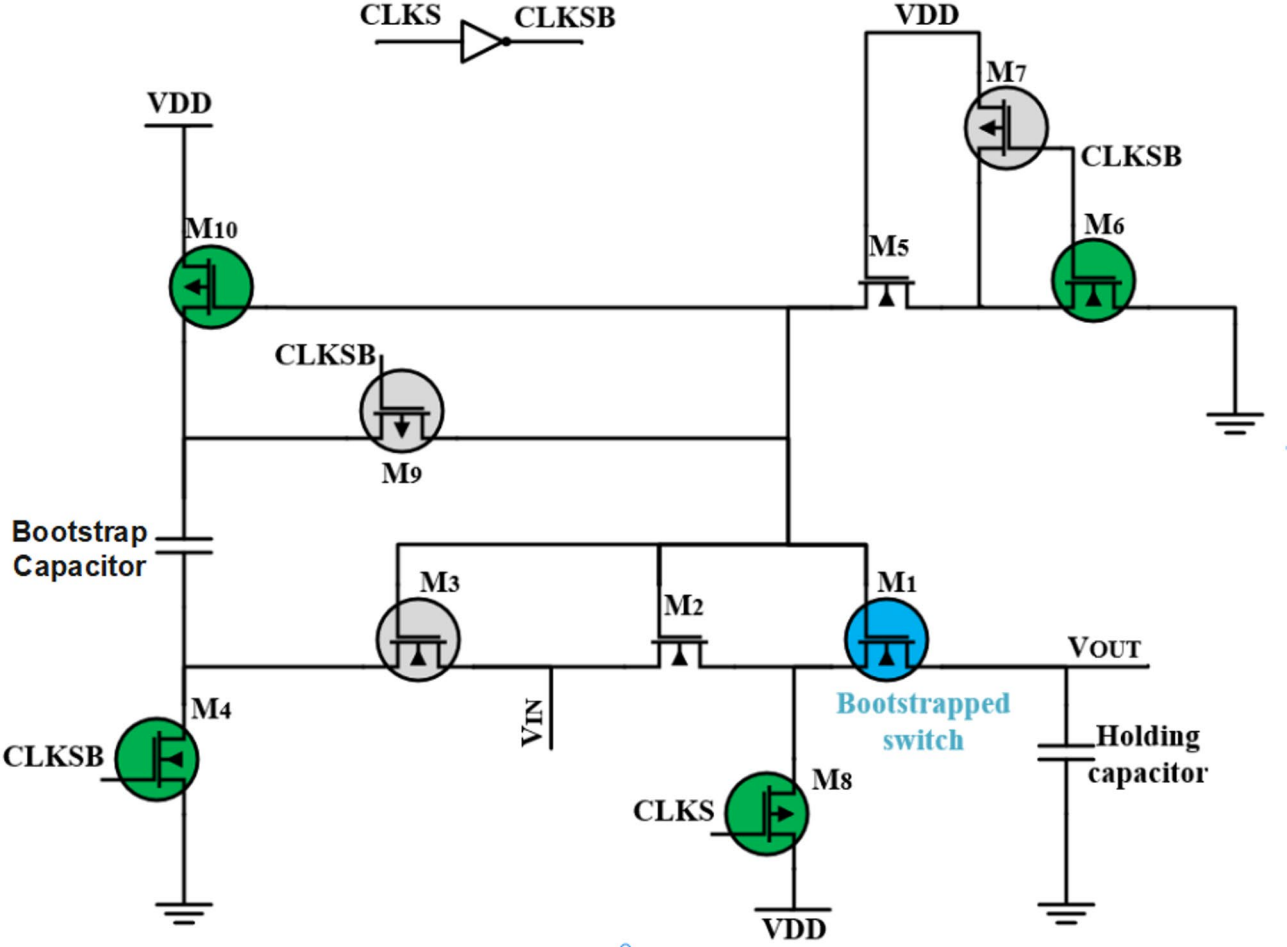
The proposed high-isolation CMOS bootstrapped switch schematic.

The schematic of the high-isolation CMOS bootstrap S/H switch is shown in Fig. 2. Two MOS transistors M7 and M8 have been added to the conventional bootstrapped switch to enhance the performance. The S/H operation is separated into two phases, the sampling phase and the holding phase.

**Fig. 3:**
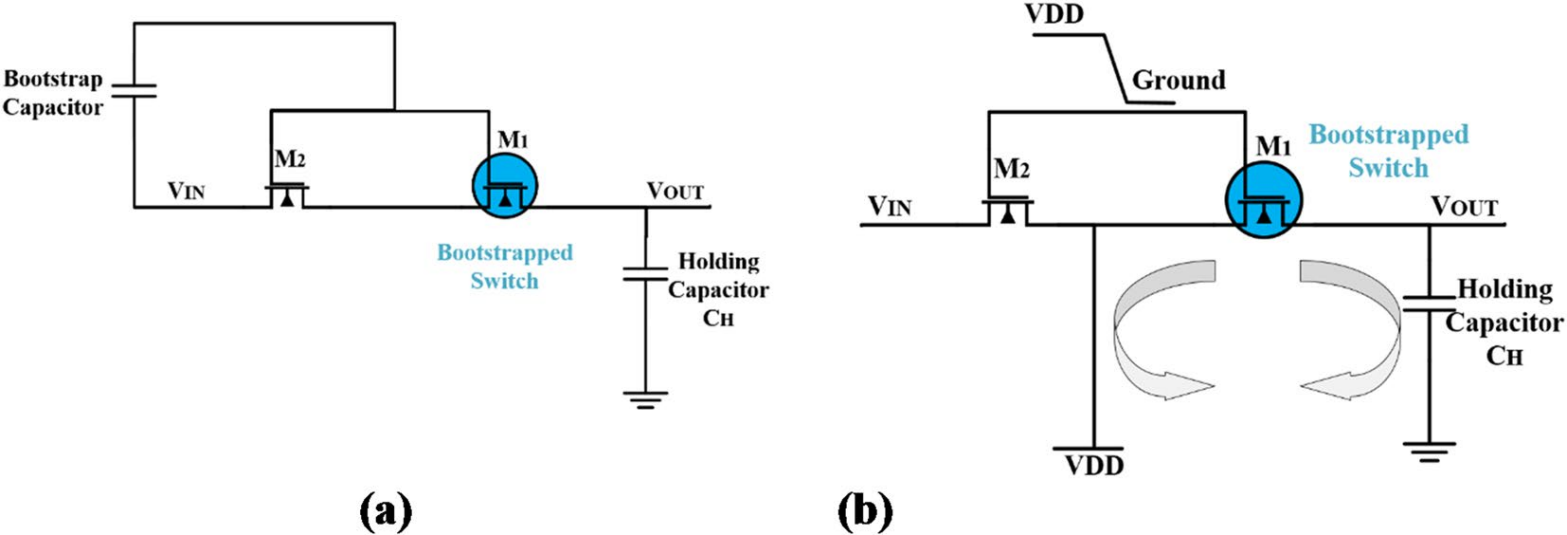
The phases of the proposed bootstrapped switch (**a**) the sampling phase, and (**b**) the holding phase.

In the sampling phase, the CLKS is high and CLKSB is low, M3, M7, M9 transistors are “ON” and M4, M6, M10 transistors are “OFF”, consequently the S/H circuit is shrunk as shown in Fig. 3a. To maintain the voltage VGS of the bootstrap switch MOS M1 constant, the stored charge on the bootstrap capacitor is kept fixed by connecting M3 and M9. Also, to enhance the linearity of the overall ADC, PMOS M7 is inserted to create a second path to ensure that the gate voltage of the bootstrap switch M1 is constant at the pull-up supply voltage.

In the opposite, in the holding phase, the CLKS is low and CLKSB is high, M4, M6, M10 transistors are “ON” and M3, M7, M9 transistors are “OFF” as shown in Fig. 3b. In this phase, the bootstrap capacitor stores charge from the supply voltage V_DD_ through M4 and M10. In addition, an isolation between the gate of M1 and the rest of the S/H circuit is attained by M3 and M9.

Simultaneously, the gate terminal of the sample switch M1 is connected to ground by using M6 to turn off M1. Consequently, the main charge injection process depends only on the input signal. The source terminal of the sampling switch M1 is connected to the supply voltage VDD through M8 which keeps the charge injection constant. Also, a cascode scheme constructed by M2 and M5 is direct connected to M1 and M6, respectively, to increase the lifetime and avoid stress of MOS transistors.

In the proposed high-isolation CMOS bootstrapped S/H switch, the non-linearity error gets better by adding NMOS switch M8. Then even if the input signal varies, the charge injection doesn’t change.

### Proposed double-tail dynamic comparator

**Fig. 4:**
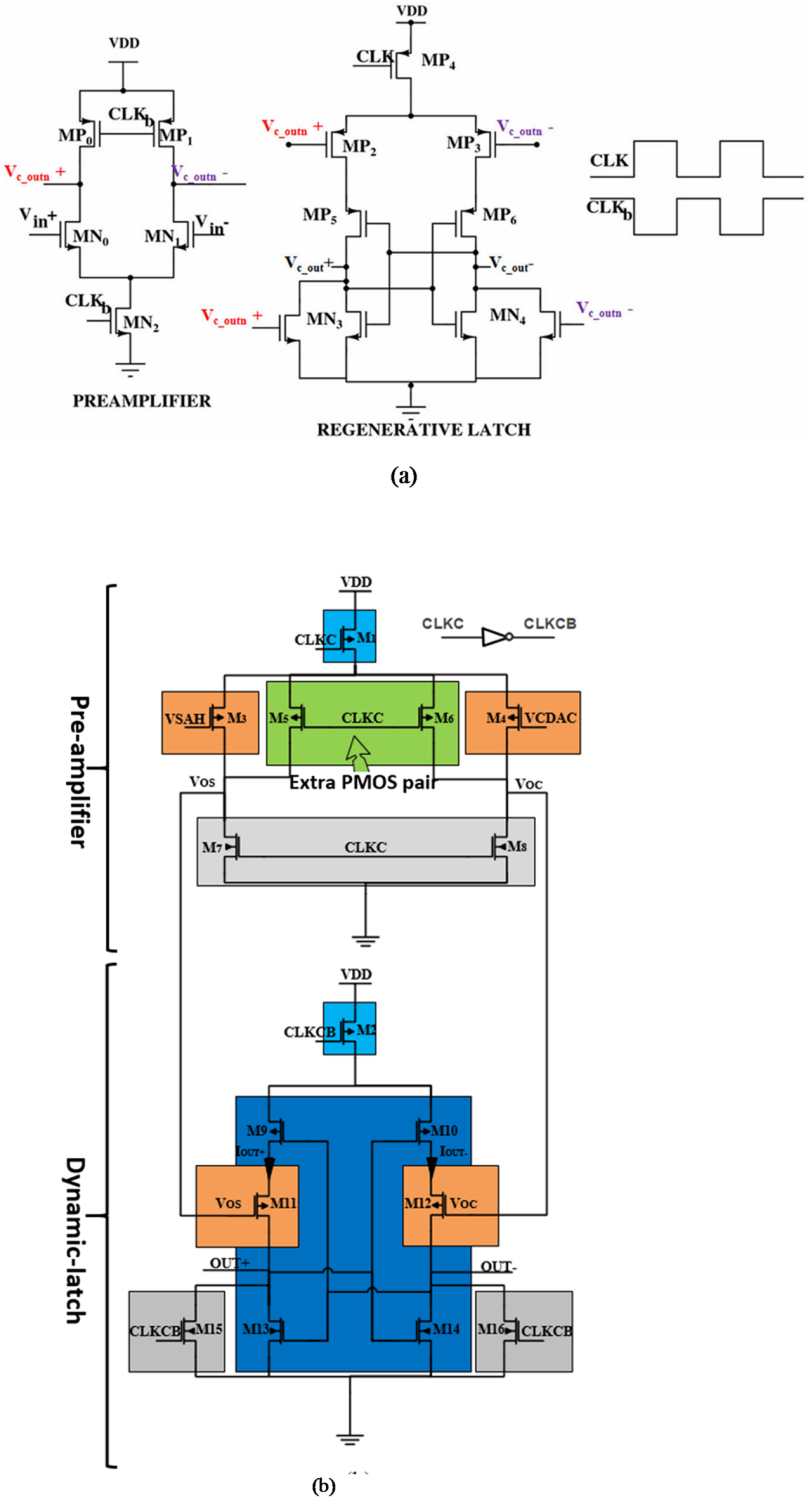
A double-tail dynamic comparator (**a**) the conventional^29^. (**b**) the proposed.

The comparator is the second building block in the architecture of the SAR-ADC, which is responsible for detecting which input terminal is greater than the other terminal; the input terminal of the S/H output voltage or the input terminal of CDAC output voltage. Generally, the SAR-ADC executes several comparisons to obtain the complete digital code. These comparisons consume most of the SAR-ADC power. Thus, the comparator is considered a big source of power consumption. For biomedical applications, the power consumption is a critical challenge. Until now the research works have been proposed dynamic latch comparators trying to overcome the great power consumption, preventing kickback noise, and reducing the input-referred offset voltage, which affect the accuracy and linearity performance of the SAR-ADC^15^. In addition, meta-stability is a phenomenon that occurs in the dynamic comparator due to the internal parasitic capacitor and the mismatch between transistors. Among the different types of the dynamic latch comparator, the double-tail dynamic comparator has less processing time and enables a low current to follow in the pre-amplifier stage which leads to low power consumption, and higher linearity performance compared to the traditional dynamic comparator^16^. As shown in Fig. 4a, the conventional two-stage double-tail dynamic comparator that includes the pre-amplifier stage, dynamic latch stage, and a double-tail transistor is the most preferred type used due to their high performance and low-power efficiency^29^. A double-tail transistor is an essential additional part in the dynamic comparator to reduce the consumed power. Also, the pre-amplifier stage is added to reduce the input-referred offset voltage^14–16^. While a high current is generated in the latch stage, which reduces the delay of the latch and enhances the meta-stability phenomenon.

We have proposed a double-tail with extra pair PMOS transistors dynamic comparator as shown in Fig. 4b. The proposed comparator reduces the kickback noise and input referred offset voltage. The proposed double-tail dynamic comparator consists of the dynamic pre-amplifier (M3, M4, M7, and M8) with extra pair PMOS transistors (M5 and M6), the dynamic latch (M9: M16), and the double-tail transistors (M1 and M2) as shown in Fig. 4b.

**Fig. 5:**
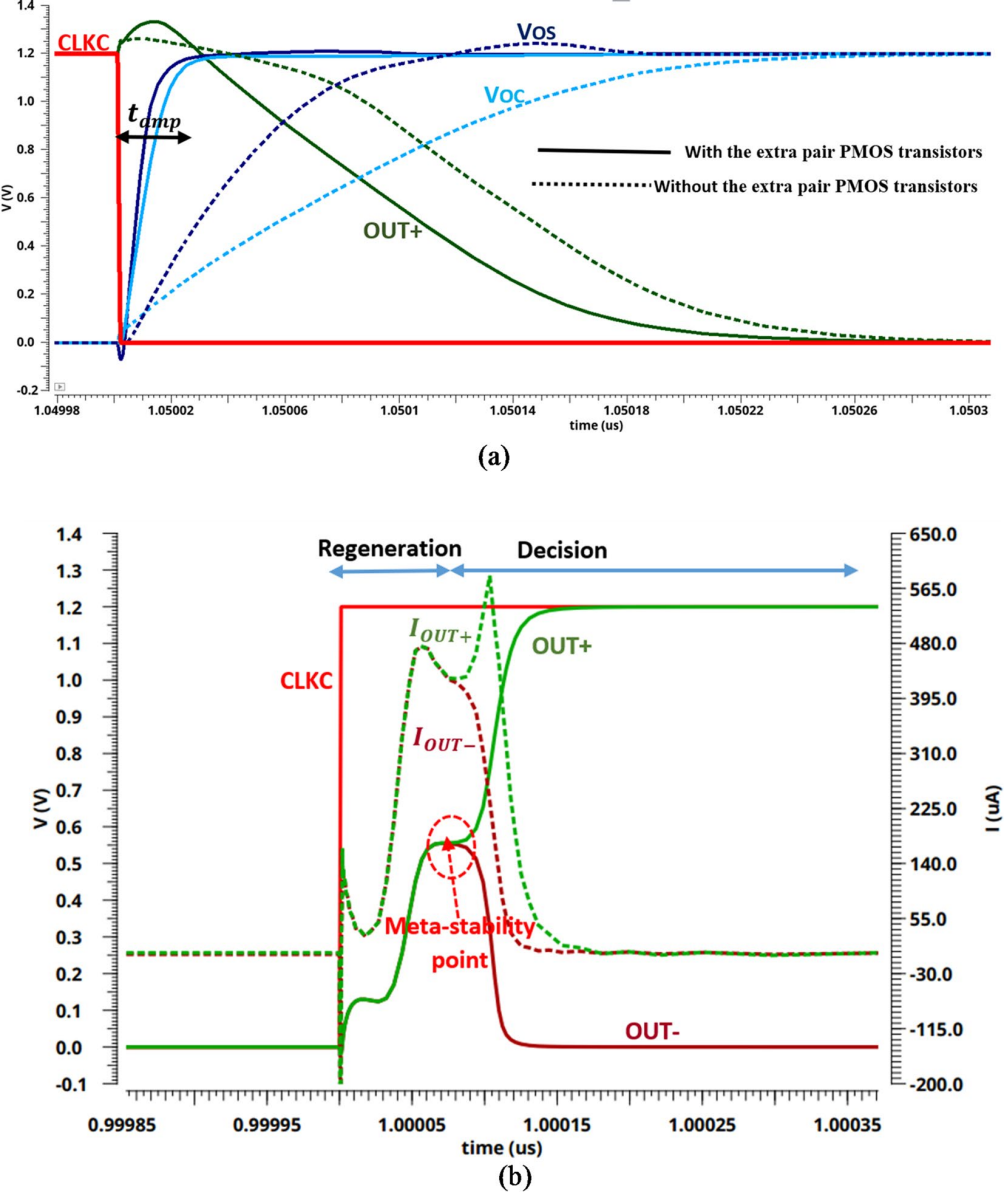
Transient simulations of the proposed dynamic comparator for input voltage difference of _Vin = 5 mV, Vcm = 0.6 V, and VDD = 1.2 V. (**a**) The operation of comparator when CLKC is low. (**b**) The operation of comparator when CLKC is high.

The operation for the proposed comparator is divided into three phases, as illustrated in Fig. 5a (reset phase) and Fig. 5b (regeneration phase and decision phase). Figure 5a shows the output nodes of pre-amplifier (VOS and VOC) and the output node of latch OUT + with and without the extra pair PMOS transistors (M5 and M6) during the reset phase. As expected, without the extra pair PMOS transistors (M5 and M6), there is a huge transient time for the output node voltages (VOS and VOC) to reach steady state. It should be noted that, as the switching time increases, the preamplifier energy increases. Figure 5b shows the output node voltage of the latch stage (OUT + and OUT-) and the currents of these nodes during the regeneration and decision phases.

During the reset phase, when CLKC goes from high to low, tail transistor M1 is on, which permits the differential branches of the pre-amplifier stage to start charging from ground to V_DD_, and the extra pair PMOS transistors (M5 and M6) help speeding the charging process by enables a charge path between the parasitic capacitors in the output nodes (VOS and VOC) and V_DD_ as shown in Fig. 5a. In the same time, the reset transistors (M15 and M16) in the latch circuit discharge the output nodes (OUT + and OUT-) to ground.

In the regeneration phase, when CLKC goes from low to high, tail transistor M2 is on, then the two output nodes, OUT + and OUT- are quickly pulled up from 0 to V_DD_. As well, the reset transistors (M7 and M8) in the pre-amplifier stage are turned on and permit the VOS and VOC nodes to discharge through the parasitic capacitors. Actually, the latch stage is still off until the pre-amplifier output nodes (VOS and VOC) get discharged enough to turn on the input latch transistors (M11 and M12). While in the decision phase, M11 and M12 are turned on; if VSAH is greater than VCDAC, the Voc discharges faster than the Vos. Therefore, the source current of M11 (IOUT+) is greater than the source current of M12 (IOUT-). Consequently, the output node voltage OUT + is charging faster than the output node voltage OUT-, as shown in Fig. 5b. Thereafter the transistor M9 will be turned on initiating the latch decision because of the back-to-back CMOS inverters (M9, M13, and M10, M14). As a result, the output node voltage OUT + will be pulled up to VDD, and the output node voltage OUT- will be discharged to ground, and vice versa as shown in Fig. 5b.

The SAR-ADC devices dissipate their instantaneous power in microwatts (µW), which is the total of two components: static power, mostly from subthreshold leakage and results from the transistor not fully shutting off, and dynamic power, which depends on the frequency of circuit activity^23^. The tiny leakage current that flows with the gate in a DC or steady state was one of the primary benefits of the dynamic comparator. On the other hand, dynamic power is consumed during the gate’s transition, which is a rising or falling clock signal^24^. It is considered the majority of the power consumed. The increase in size of the preamplifier transistors has dropped the offset voltage and the kickback noise due to enhancing the gain of the preamplifier stage and reducing the value of parasitic capacitors of input transistors. On the other hand, improving the size of the latch transistors is necessary to reduce the regeneration phase’s delay time, which raises the dynamic comparator’s speed. Despite the fact that transistor size affects power consumption. Therefore, selecting the optimum size for the design is a challenging task that aims to achieve a balance of performance between speed, noise, input offset voltage, and power consumption.

The dynamic power consumption of the proposed comparator ($\:{\text{P}}_{\text{c}\text{o}\text{m}\text{p}}$) is composed with two terms, the dynamic pre-amplifier power ($\:{\text{P}}_{\text{p}\text{r}\text{e}-\text{a}\text{m}\text{p}}$) and the dynamic latch power ($\:{\text{P}}_{\text{l}\text{a}\text{t}\text{c}\text{h}}$).

In the regeneration phase, the CLKC signal is rising, and the most power is consumed by the latch stage. the two parasitic capacitors at the output nodes OUT + and OUT- are charged by using the transistors M11 and M12 operated in the triode region which acts as linear resistors, until reaching the threshold voltage of transistors M13 and M14, respectively as shown in Fig. 5b. Otherwise, in the decision phase, one of the output parasitic capacitors charges to the supply voltage (VDD), and the other one discharges to ground^35^.

Also, during the reset phase; the CLKC signal is falling, and a huge amount of power is consumed by the pre-amplifier stage. The power consumption depends on the parasitic capacitor at points (VOS and VOC) and the falling current at these points, which is a function of the differential source-drain voltage of M3 ($\:{V}_{{SD}_{{M}_{4}}})$ or M4 ($\:{V}_{{SD}_{{M}_{4}}})$. The proposed comparator power is expressed as follows:

3 $$\:{P}_{comp}=\frac{1}{{T}_{CLKC}}{V}_{DD}\left[{C}_{OUT+}\left({V}_{DD}+{V}_{{tn}_{M13}}\right)\right]+\:\:\frac{1}{{T}_{CLKC}}{\int\:}_{0}^{{t}_{amp}}{V}_{DD}{I}_{M1}dt$$

4 $$\:{P}_{comp}=\frac{1}{{T}_{CLKC}}{V}_{DD}\left[{C}_{OUT+}\left({V}_{DD}+{V}_{{tn}_{M13}}\right)\right]+{(I}_{M3}+{I}_{M5}+{I}_{M4}+{I}_{M6}\left){t}_{amp}\right]$$

Where $\:{t}_{amp}$ is the transient time with falling clock signal.

**Fig. 6:**
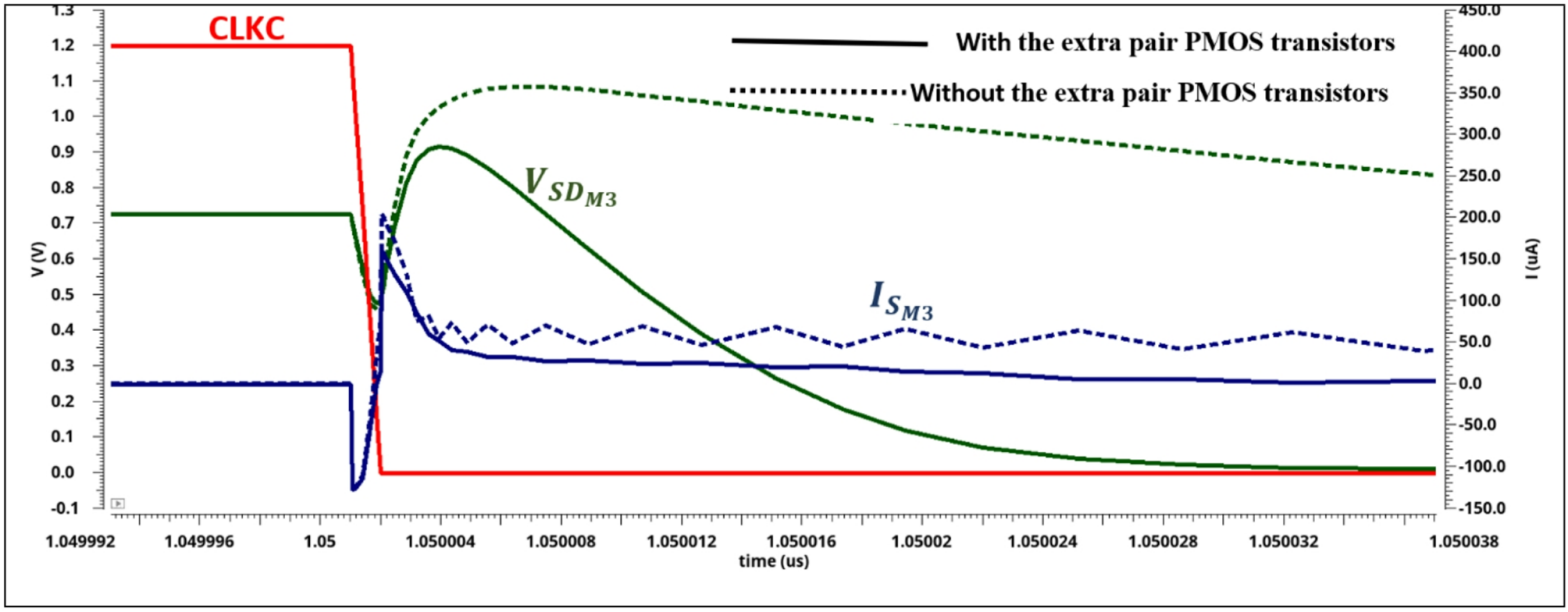
The simulated transient time of the reset phase in the proposed dynamic comparator.

In the above equations, the power consumption depends strongly on the current of the input differential pair and the extra pair PMOS transistors. Figure 6 shows the simulated transient time (tamp) of the input current transistor ($\:{I}_{{S}_{M3}}$) and the differential voltage input transistor between source and drain ($\:{V}_{{SD}_{M3}}$) with and without two transistors (M5 and M6). By adding the extra pair PMOS transistors (M5 and M6), the differential source-drain voltage of (M3 and M4) is decreased. Also, the switching falling time has been diminished by adding a second path to charge the parasitic capacitor quickly at the output pre-amplifier voltage nodes (VOC and VOS), based on Fig. 5.a. The input threshold voltage point of the pre-amplifier is decreased by falling the clock signal, which in turn varies the source-drain current of the two input transistors, thus dropping its transient time and the total energy of the circuit. So, the total load parasitic capacitor at the output pre-amplifier voltage nodes (VOC and VOS) is decreased. The comparator power can be approximated as follows:

5 $$\:{P}_{comp}=\frac{1}{{T}_{CLKC}}{V}_{DD\:}\{\:{C}_{OUT+}\left({V}_{DD}+{V}_{{tn}_{M13}}\right)+{C}_{{V}_{OC}}\left({2\:V}_{DD}\right)\:\:\}$$

**Fig. 7:**
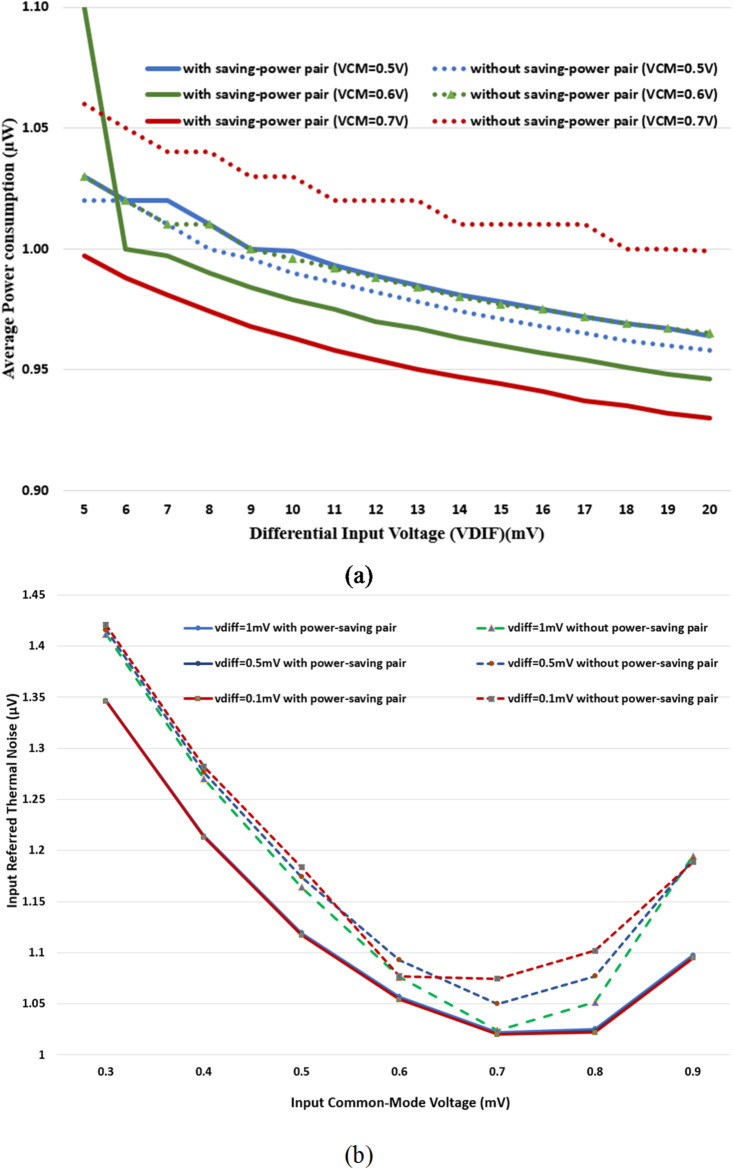
The proposed comparator with and without the extra pair PMOS transistors (**a**) The average power consumption versus the differential input voltage (**b**) The input-referred thermal noise versus the input common-mode voltage.

By adding the extra pair PMOS transistors, the charging process of the output nodes VOS and VOC is speeded up when the CLKC is low. Consequently, the current through the input transistors M3 and M4 is decreased compared with the same currents without the extra pair PMOS transistors M5 and M6. Thus, the total average power consumption is reduced, as shown in Fig. 6. With different common mode voltages (VCM), the impact of the differential input voltage (VDIF) on the total average power consumption is shown in Fig. 7(a). In addition, when VCM is higher than VDD/2, the extra pair PMOS transistors enhances the energy efficiency of the dynamic comparator. A minimum average power consumption is obtained for the proposed dynamic comparator. Also, the comparator’s input-referred thermal noise can’t be neglected in the design. So, Fig. 7(b) shows the input-referred thermal noise versus input common-mode voltage with differential input voltage for both cases: (1) without a power-saving PMOS pair and (2) with the power-saving PMOS pair. At the minimum differential input voltage, the input-referred thermal noise is decreased for the proposed dynamic comparator. The extra pair PMOS transistors supports increasing the energy efficiency of the proposed comparator, making it suitable for biomedical applications.

The reset signal for the comparator is boosted to minimize any memory impact between two comparisons, especially when there is a large step change in the comparator input. During the reset phase, when the clock is falling, large voltage variations exist on the output pre-amplifier nodes (VOS and VOC) are fed back into the differential input nodes of the comparator through the parasitic capacitor of the gate and drain terminals (Cgd). These feedback variations on the input terminals are usually called kickback noise^14^. The p-type input pair transistors are employed in the pre-amplifier to reduce this noise.

**Fig. 8:**
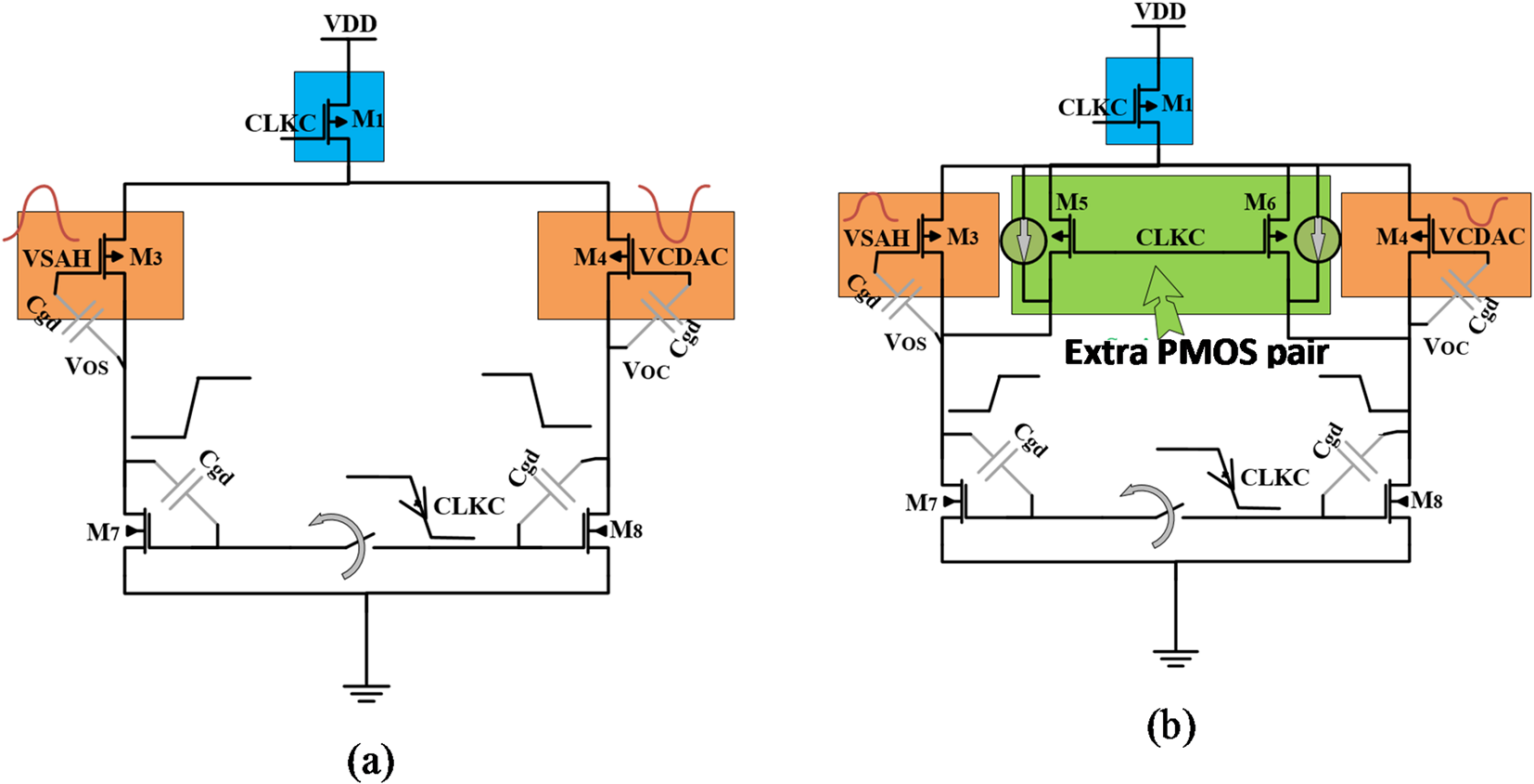
The parasitic models of the kickback noise in the pre-amplifier stage (a) without (b) with the extra pair PMOS transistors.

The parasitic models of the kickback noise in the pre-amplifier stage without and with the extra pair PMOS transistors are shown in Fig. 8. In the proposed scheme, the core idea is to add another path to quickly discharge the leakage current through the parasitic capacitance of the extra pair PMOS transistors and isolate the input terminals of the differential-pair transistors from the output nodes (VOS and VOC) to reduce the feedback variations and consequently the kickback noise.

**Fig. 9:**
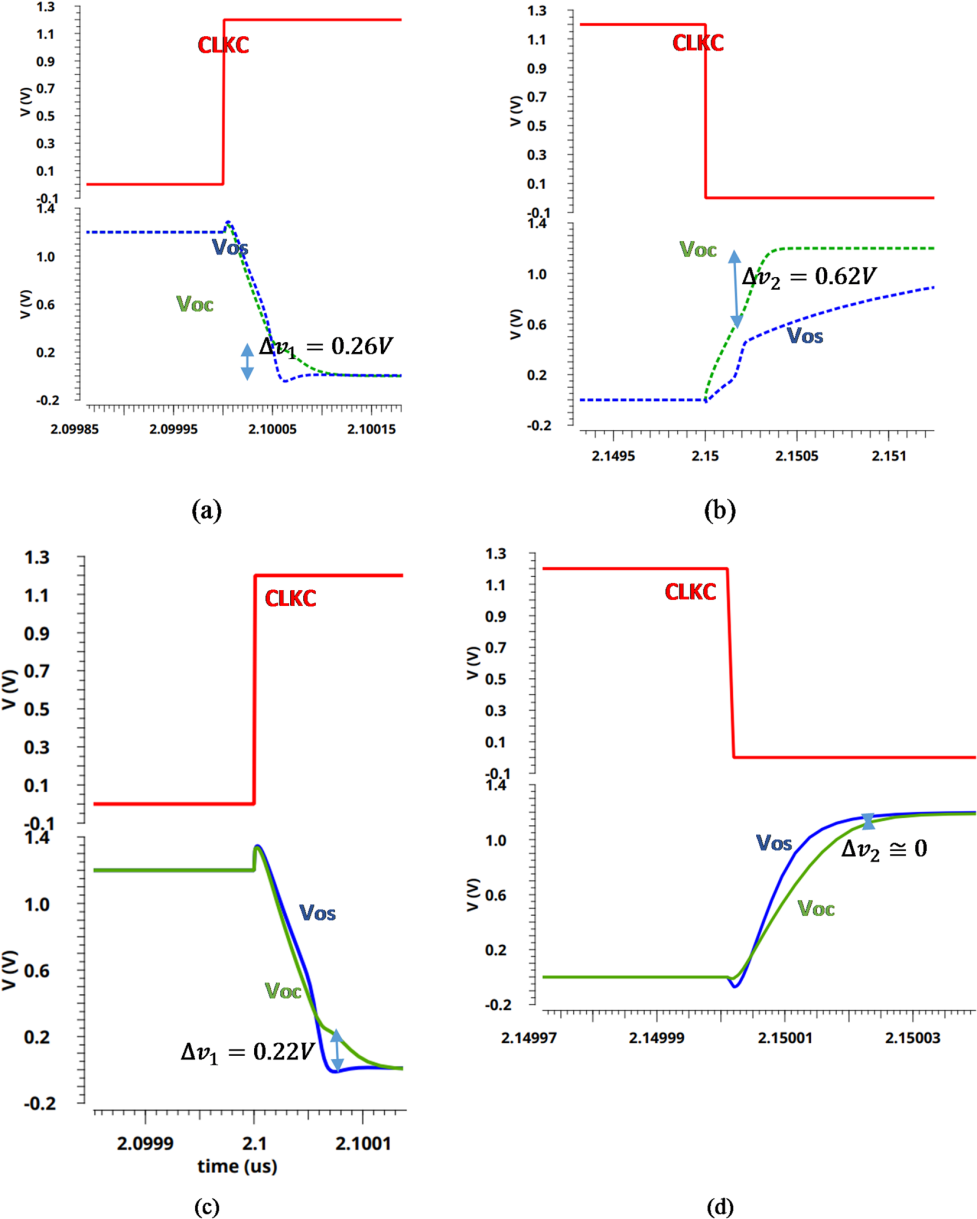
The variation voltage at drain of input transistors (**a**) regeneration phase (**b**) reset phase -without the extra pair PMOS transistors- (**c**) regeneration phase (**d**) reset phase -with the extra pair PMOS transistors.

As shown in Fig. 9 (a, b), the variation of Vos and Voc nodes ($\:\varDelta\:{v}_{1}$) will couple back to the gate terminal of input transistors (M3, M4) through the parasitic capacitor, resulting in the kickback noise in the regeneration and resetting phase, respectively. This variation in the regeneration phase ($\:\varDelta\:{v}_{1}$) is decreased due to a decrement in the clock feedthrough and charge injection of the extra pair PMOS transistors, as simulated in Fig. 9 (c). During the resetting phase, as simulated in Fig. 9 (d), the Vos and Voc voltages are quickly pulled up to VDD by shorting switches the extra pair PMOS transistors, which results on a reduction in the variation of Vos and Voc nodes ($\:\varDelta\:{v}_{2}$).

It should be noted that the proposed double-tail dynamic comparator decreases the impact of feedback noise on the input terminals. As a result, the variation of output nodes, kickback noise, and input offset voltage are decreased. Moreover, according to the definition of ENOB of SAR-ADC^4,24^, as the noise of distortion decreases, the ENOB increases. In the post-results and comparison section, the ENOB is improved by up to 0.3 bit while the SNDR is enhanced by more than 2 dB.

### Capacitive DAC

In this subsection, the proposed digital-to-analog converter DAC is discussed. The binary-weighted capacitive DAC (CDAC) exhibits lower energy dissipation with higher linearity performance than the resistor-based DAC, making it the typically used DAC for biomedical applications^1^.

Unfortunately, a large number of capacitors are required; the total number of capacitors is $\:{2}^{N}{C}_{u}$, where $\:{C}_{u}$ is the technology unit capacitor. As a result, a large area is occupied by the binary-weighted CDAC, hence the cost of the ADC is increased. Different CDAC schemes have been proposed for saving energy with less area^3,21^. It is noticeable that the bridge CDAC structure can decrease the total area and cost. The bridge CDAC achieves higher energy saving and less area as compared with the conventional binary-weighted CDAC. In addition, the linearity is enhanced as compared with the dual split CDAC^25^.

**Fig. 10:**
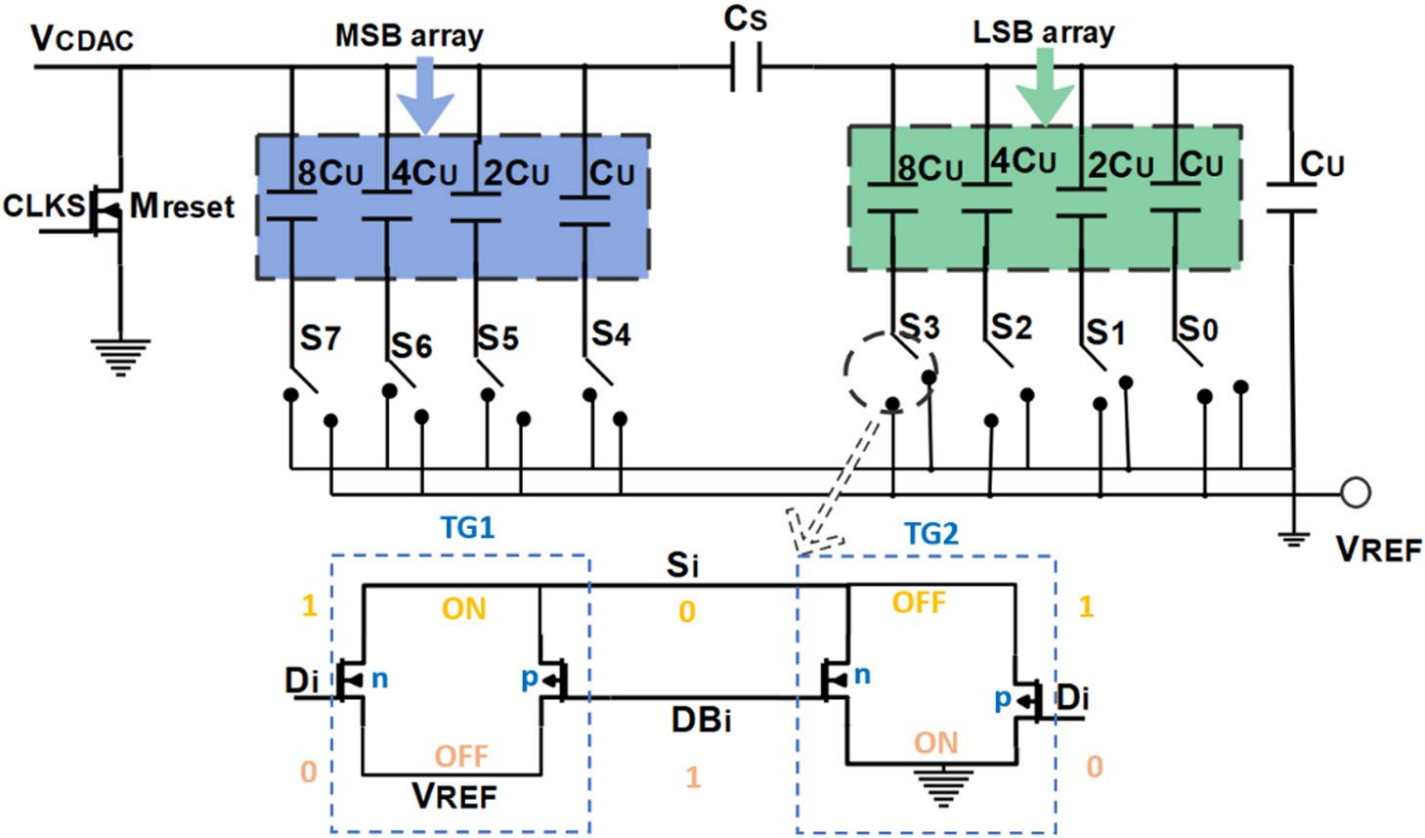
The proposed bridge capacitive DAC.

The bridge capacitive array is split into two subarrays, the MSB array (M-bits) and the LSB array (L-bits), by a split capacitor (CS), as shown in Fig. 10. The calculation of the split capacitor is based on Eq. (6)^3^. The bridge CDAC involved eight multiplexers (2 to 1) perform as switch arrays, which are designed by two transmission gates.

6 $$\:{C}_{S}=\frac{\sum\:{C}_{LSB\:array}}{\sum\:{C}_{MSB\:array}}=\frac{{C}_{TLSB}}{{C}_{TMSB}}$$

As shown in Fig. 10, the digital output bits of SAR logic ($\:{D}_{\text{i}}\:,i\to\:0:7$) are used to control the switching array by selecting one of the transmission gates to work. If the D_i_ selection control is “high,” the transmission gate (TG1) is activated and then the reference voltage ($\:{V}_{REF}$) will be connected to the input of the CDAC. Similarly, if the D_i_ selection control is “low”, the transmission gate (TG2) is activated, and then the ground will be connected to the input of the CDAC.

During the sampling phase, the $\:{V}_{CDAC}$ is connected to the ground through $\:{M}_{reset}$, as shown in Fig. 10, to release the total charge of the capacitors. After the sampling phase, the $\:{M}_{reset}$ is turned off and the MSB capacitor is connected to $\:{V}_{REF}$, where the regeneration phase starts in the comparator. The output of the bridge CDAC is derived from Eq. (7), so the initial $\:{V}_{CDAC}$ is equal to $\:{V}_{REF}/2$, which is fed back to one input of the comparator.

7 $$\:{V}_{CDAC}=\left\{{V}_{{CDAC}_{MSB}}+{V}_{{CDAC}_{LSB}}=\left\{\begin{array}{c}{V}_{{CDAC}_{MSB}}=\frac{\sum\:_{i=0}^{M-1}{D}_{L+i}{2}^{i}{C}_{u}}{{C}_{TOT}}{V}_{REF}\\\:{C}_{TOT}=\frac{{C}_{S}{C}_{TLSB}}{{C}_{S}{+C}_{TLSB}}+{C}_{TMSB}\\\:{V}_{{CDAC}_{LSB}}=\frac{{C}_{S}}{{C}_{S}{+C}_{TLSB}}\frac{\sum\:_{i=0}^{L-1}{D}_{i}{2}^{i}{C}_{u}}{{C}_{TLSB}}{V}_{REF}\end{array}\right.\right.$$

If $\:{V}_{SAH}>\:{V}_{CDAC}$, the comparator results one, then the MSB switch is still connecting to $\:{V}_{REF}$; otherwise, if $\:{V}_{SAH}<\:{V}_{CDAC}$, the comparator results zero, then the MSB switch is connected to the ground through switch S7. This operation is repeated at each starting edge of CLKC for each capacitor until all digital code is stored in the SAR logic register.

Table 1 shows the calculated value, the simulation value, and the percentage error of the set of digital inputs. The worst percentage error of bridge CDAC is smaller than 0.5%.

**Table 1:** Bridge CDAC simulation results.

Digital input								Calculated (V)	VDAC (V)	Error %
D7	D6	D5	D4	D3	D2	D1	D0			
0	0	0	0	0	0	0	0	0	0.000405	$\:\cong\:$0
1	0	0	0	0	0	0	0	0.6	0.598	0.33
1	0	0	0	1	0	0	0	0.637	0.63515	0.29
1	1	0	0	0	0	0	0	0.9	0.896	0.44
1	1	0	0	1	1	0	0	0.956	0.952	0.418
1	1	1	1	0	0	0	0	1.125	1.1198	0.46
1	1	1	1	1	1	0	0	1.18	1.175	0.42
1	1	1	1	1	1	1	1	1.195	1.193	0.167

### The synchronous SAR-logic block

**Fig. 11:**
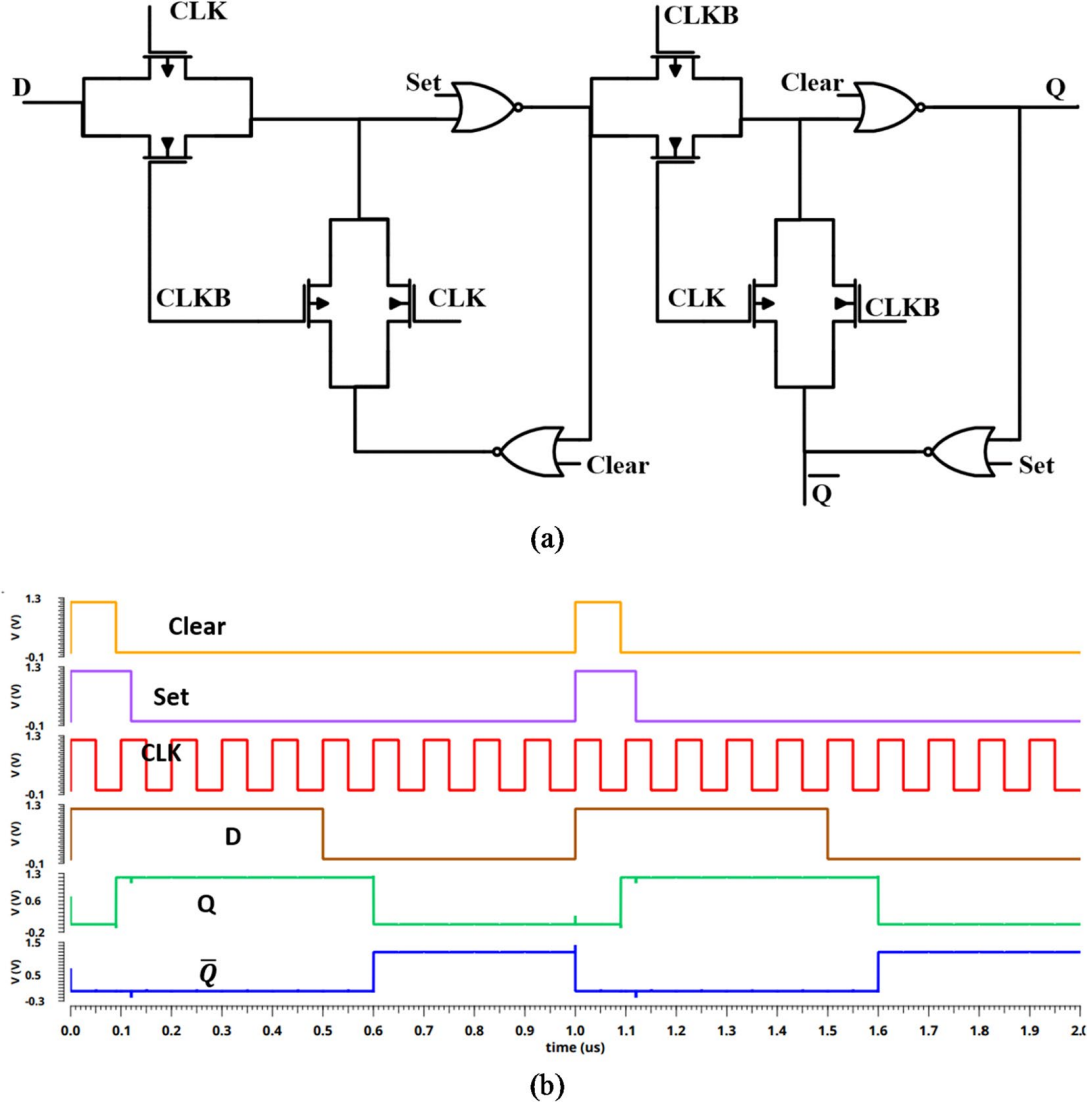
The schematic for set-clear DFF (**a**) circuit^22^. (**b**) the time sequences.

In this subsection, the proposed synchronous SAR logic register is discussed. Between the comparator and CDAC, the SAR logic is placed to generate the output code according to the comparator’s decision. It is important to note that significant power is consumed by the SAR logic because of the large number of registers employed in the implementation, making it dominate the total power of SAR-ADC. The synchronous SAR logic is split into two branches, the sequencer cascaded registers branch and the code registers branch which are heavily based on D-flip flops (DFFs). A (2 *N* + 1) set-clear DFF is employed in this scheme. Figure 11 illustrates the schematic and timing sequence for the proposed set-clear DFF. To achieve minimum power consumption and less active area while maintaining low leakage current, we have proposed four transmission-gates and four NOR gates to implement the DFFs with minimum size^22^, as shown in Fig. 11 (a). When clear is one, the Q-output and $\:\stackrel{-}{Q}$-output will be zero. Also, it can be seen that as clear is falling and set is rising, the Q-output will be one. While, when the clear and set are zero, and the CLK is rising, the Q-output will transfer the input data, as shown in Fig. 11 (b).

**Fig. 12:**
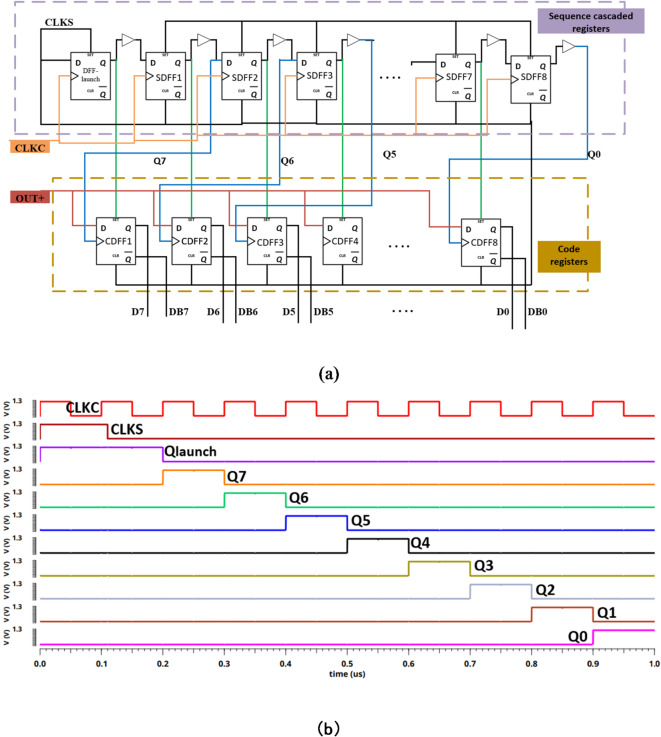
The block diagram of the proposed synchronous SAR logic (**a**) the sequencer cascaded registers branch with the code registers branch. (**b**) the output of the sequencer cascaded registers branch.

The proposed SAR logic register block diagram and timing diagram are depicted in Fig. 12. As mentioned before, a sequencer cascaded registers branch and a code registers branch act together to form the SAR logic as shown in Fig. 12 (a). The operation timing diagram of our proposed register branch is presented in Fig. 12 (b). The sequence cascade registers branch is responsible for well arrange the initiating time of the code register branch. Firstly, during the sampling phase, the launch DFF is triggered to set-up mode while all other DFFs are put in the reset mode by activating the clear terminal. The sequence registers output (Q7:Q0) and the digital code output (D7:D0) will be zero. As a result, the capacitor array of the DAC will be switched to ground. On the other hand, during the holding phase, in the coming rising edge of CLKC, the output of the first D-FF of the sequence cascade registers branch is one. Similarly, with every rising edge of CLKC, this operation starts again with the next D-FF and outputs one as shown in Fig. 12 (b). The code registers branch receives these outputs (Q7:Q0) and produces the appropriate output codes (D7:D0) which are then used to control the charge of the capacitive array of CDAC.

The operation of the code registers branch starts when the output of the launch-DFF is fed to the set input of the first code chain register (CDFF1); therefore, the output of CDFF1 is a forced one, where the MSB (D7) becomes one, and all other code register outputs remain zero. Therefore, a digital code of “10000000” is inserted into the switch array CDAC, resulting in VREF/2 the output of CDAC that fed in the compactor input to be compared with the output of S/H. Afterwards, the result of the comparator is stored in CDFF1, and sequentially the set input of CDFF2 is forced to one; the MSB-1 (D6) becomes one. This procedure is repeated at each rising edge of CLKC for each code register until the LSB code register. The proposed SAR logic register exhibits low power consumption which will be presented in the following section as a simulation result.

## Post-simulation results and comparisons

**Fig. 13:**
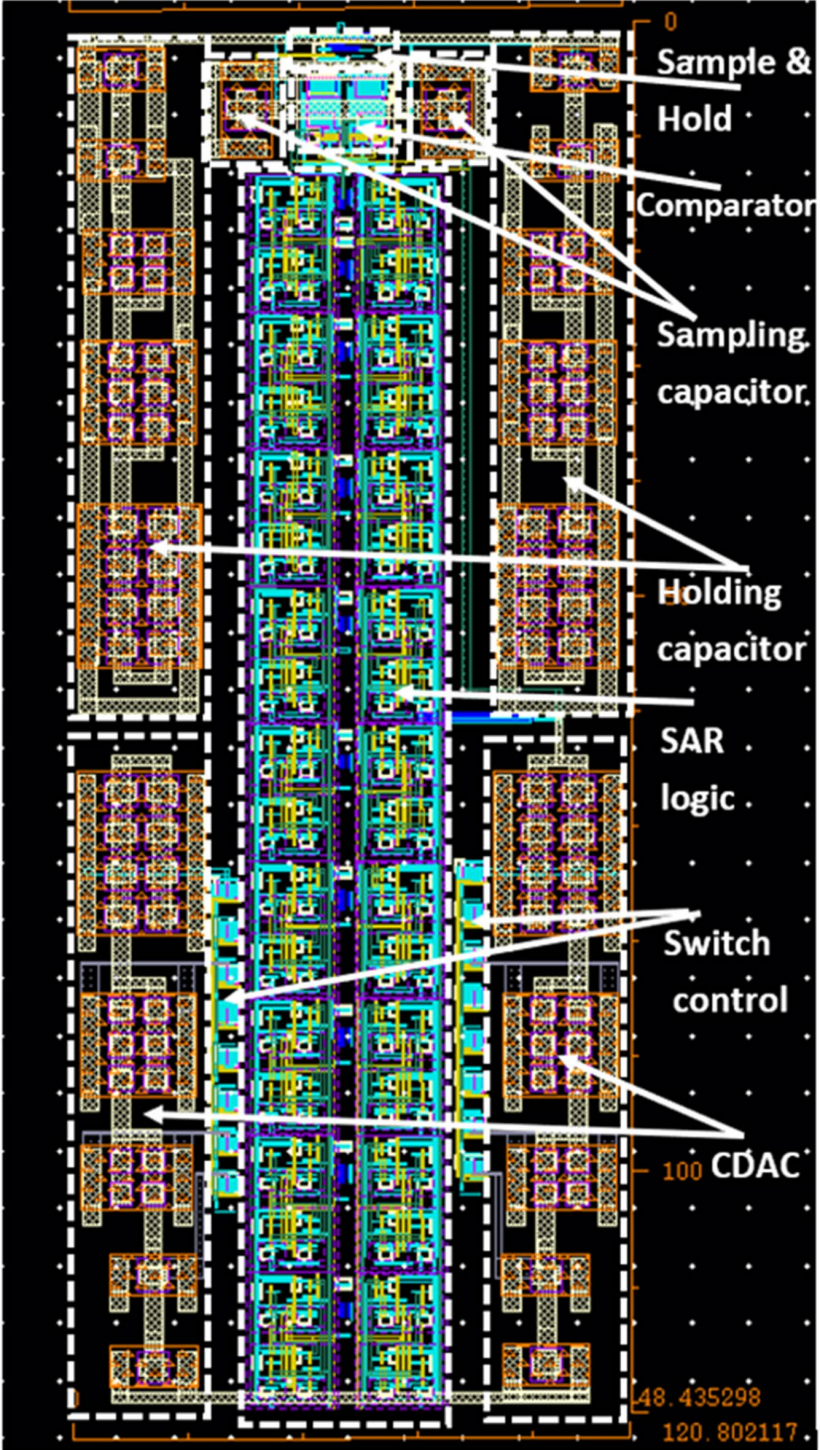
The layout of the proposed SAR ADC.

**Fig. 14:**
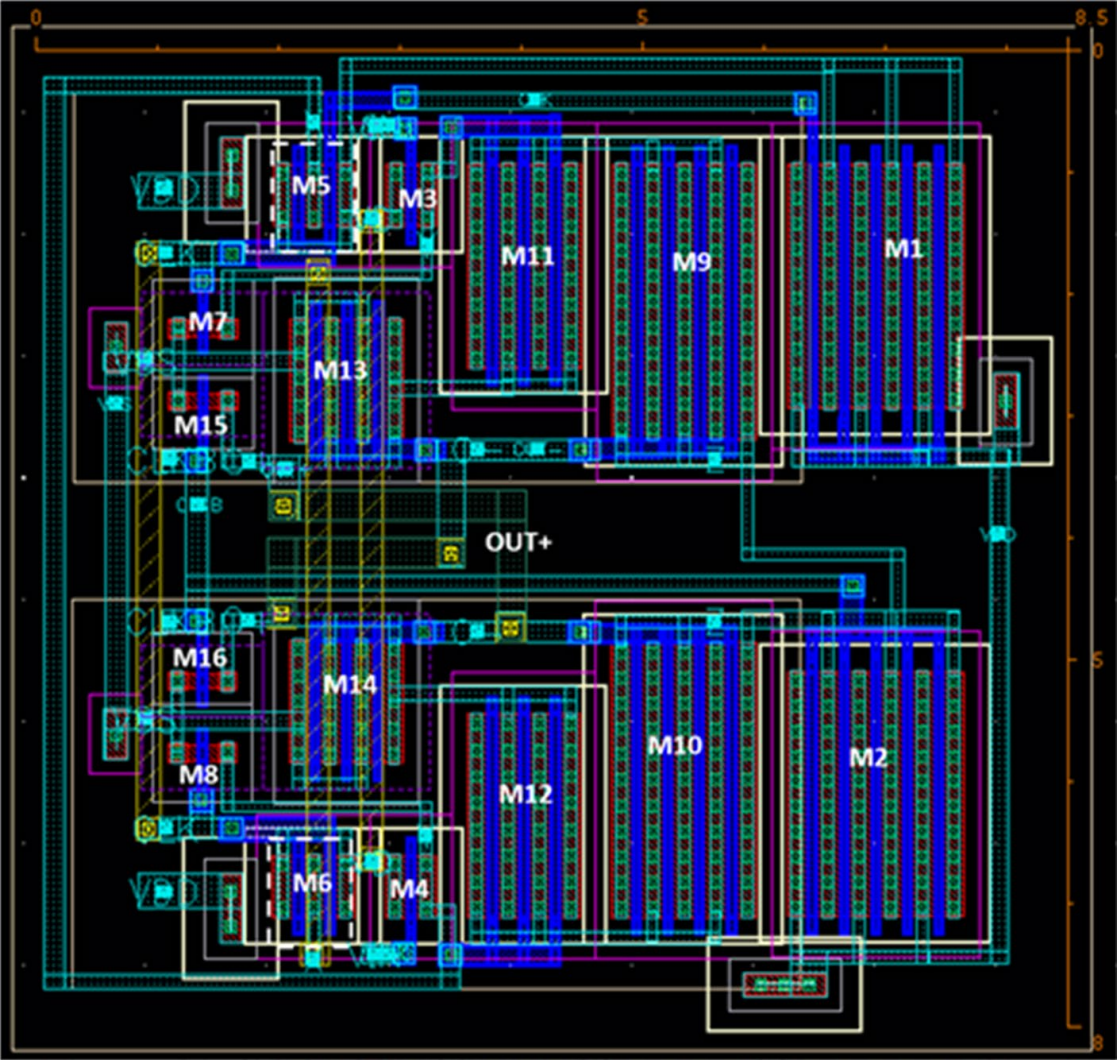
The layout of the proposed comparator.

Due to the advances in CMOS technologies with significantly low supply voltage also the advances in ultra-low power biomedical technologies, a big challenge faces the ADC designers to overcome the detection of small input voltages with minimum power consumption. This work presented 7.763-ENOB, 48.5 dB-SNDR, 53 dB-SFDR, 5.75-µW, 1-MS/s single-ended SAR ADC in 65-nm CMOS, 1.2 V supply voltage, the substrate of PMOS is connected to VDD and NMOS to ground. The complete SAR ADC including high-isolation CMOS bootstrap S/H switch, extra pair PMOS double-tail dynamic comparator, bridge capacitive array DAC, and SAR logic register, achieves 5.75 µW of power consumption and occupies an active area of 0.00585 mm^2^. As shown in Fig. 13, the layout of the proposed SAR ADC is organized as follows: the SAR control logic is placed at the middle downside of the ADC, while the capacitor array is placed at the right and left down sides. The bootstrapped S/H switch and the comparator are placed at the upper middle side, while the capacitors of the S/H sampler are placed at the right and left upper sides. Capacitor switches are placed at the right and left between the SAR control logic and capacitor array. The metal-insulator-metal (MIM) capacitor is used to implement the capacitor array of the DAC. The unit capacitor is 10 fF with an area of 2.77$\:\times\:$2.77 µm^2^. The comparator post-layout results, including parasitic, compared with the works in state-of-the-art are listed in Table 2. The proposed double-tail with extra pair PMOS transistors dynamic comparator provides power saving above 7.5% as compared with the conventional double-tail dynamic comparator at the same size of transistors, in 65 nm CMOS process. Figure 14 presents the layout of the proposed comparator with an active area of 68 µ$\:{\text{m}}^{2}$.

**Table 2:** Comparison of the proposed comparator with prior comparators.

	This work		^26^* 2024	^27^* 2023	^28^* 2023	^29^* 2023	^30^** 2022	^31^** 2021	^32^** 2020	^33^** 2019	^34^** 2018	^35^** 2018
Tech. [nm]	65	65	65	28	65	65	180	130	65	180	65	180
Supply [V]	1.2	1.2	1.2	1.8	1.1	1.2	1.2	1.2	1	1.2	1.2	1.8
Power [µW]	1.156	32	25.13	233	81.72	33.5	22	215fJ	4.8	72.2	1.7	230
Area [$\:{\text{m}\text{m}}^{2}$]	8.5$\:\times\:$8		233	–	12$\:\times\:$12	20$\:\times\:$8	16$\:\times\:$18	16.64$\:\times\:$12.98	25$\:\times\:$41	14$\:\times\:$18	12.5$\:\times\:$10	18.9$\:\times\:$16.5
Fclk [MHz]	10	500	1000	330	2500	500	100		25	500	50	500
V_*os*_ [mV]	0.125	4.86	–	3.54	11.38	8	–	11	–	7.3	–	2
Input referred Noise [µV]	1.27	0.147	657.4	102.5	750	2000	–	420	220	–	400	28
Clock-to-Q Delay [ps]	94.4	70	128.7	2400	46.5	70	292	211		268.6	1240	150
$\:\mathbf{F}\mathbf{O}\mathbf{M}$ [fJ/conv.]	0.024	0.518	–	2.77	0.676	0.893	–	3.94	–	1.757	–	1.022

*Simulated.

**Measured.

As demonstrated in Table 2, the proposed design has the least offset voltage (Vos) and low input referred noise with optimum power. For comparison, a figure of merit (FOM) is employed which relies on power consumption, speed, and accuracy as illustrated in Eq. (8). Therefore, it has intuitive significance when comparing various designs.

8 $$\:FOM=\frac{{P}_{comparator}}{{2}^{n}{F}_{clk}}\:=\:\:\frac{2*{v}_{offset}*{P}_{comparator\:\:\:}}{\:\:{V}_{dd}\:{F}_{clk}}\:\:\:\left(\raisebox{1ex}{$J$}\!\left/\:\!\raisebox{-1ex}{$conversion$}\right.\right)$$

**Fig. 15:**
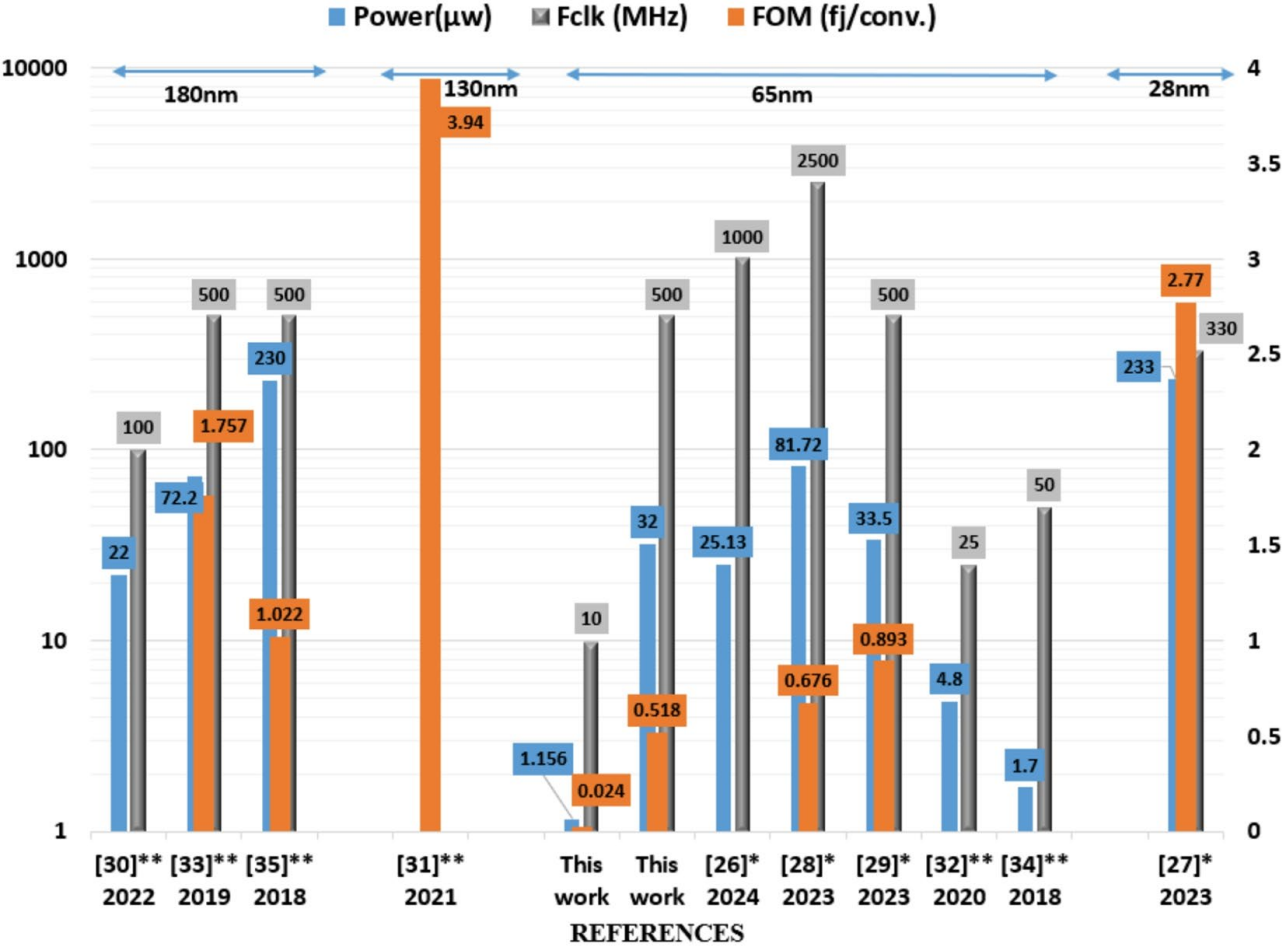
Comparison of the FOM, power consumption, and clock frequency of the proposed comparator with state-of-art comparators.

Where: $\:{P}_{comparator\:\:\:},n,\:and\:{F}_{clk}$, are the power dissipation, the number of bits (resolution), and the operating frequency of the comparator, respectively. The resolution has a direct relationship with the offset voltage. In other words, the offset voltage is equal to 0.5 LSB. Figure 15 shows the FOM, power consumption, and clock frequency comparison among ten different dynamic comparators. The FOM is very popular in dynamic comparators performance estimation. It can be noted that the proposed design has achieved the least power and area with optimum FOM. At operating frequency of 500 MHz, the proposed comparator achieves the lowest power and FOM as compared with^29,33,35^.

**Fig. 16:**
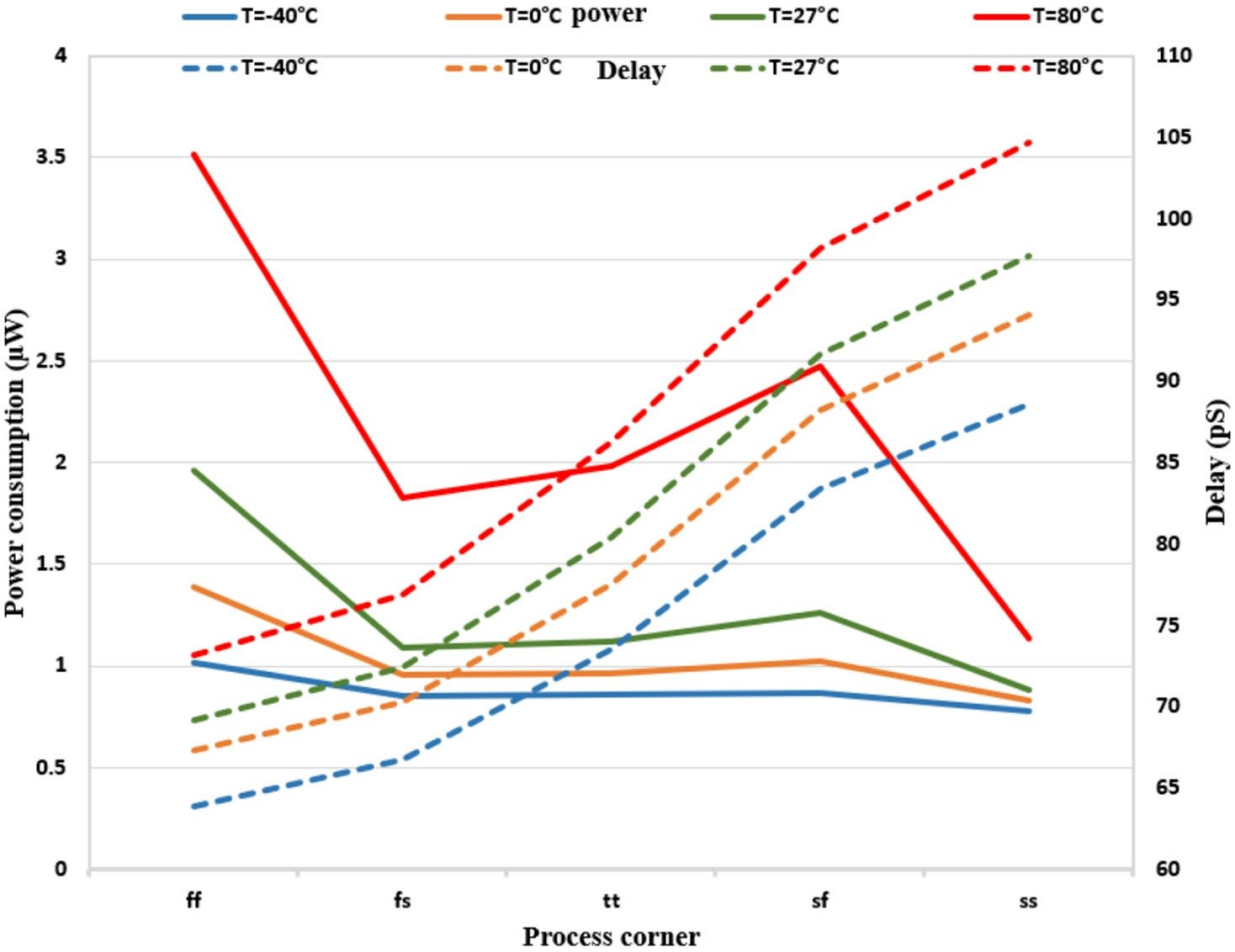
Comparison of effect of process corner variation on power consumption and delay for proposed comparator with different temperatures.

The power consumption and delay of the proposed dynamic comparator are simulated with 10 MHz clocked frequency over different process corners and temperatures, as shown in Fig. 16. Despite manufacturing corners and temperature fluctuations, It can be observed that the lowest power consumption (0.77µW in SS at -40 °C) and the highest power consumption (3.5µW in FF at 80 °C) have been attained. The careful circuit and post-result are designed to ensure the least latency of the delay cell (63 pS in FF at − 40 °C) and the maximum delay of the comparator (104 pS in SS at 80 °C) across process corners and temperature variations. The modifications in the suggested comparator are appropriate based on Fig. 15.

**Fig. 17:**
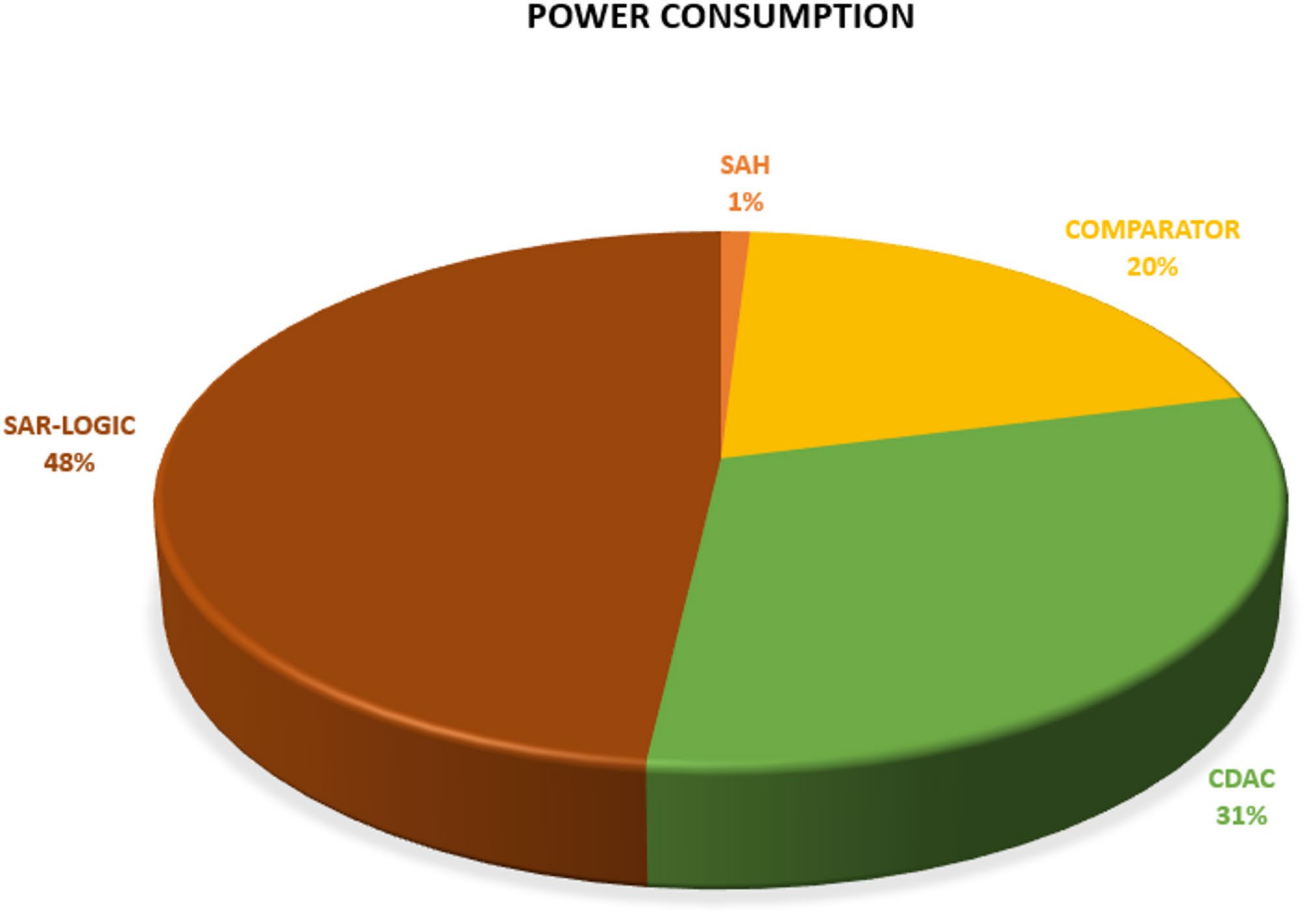
Power breakdown for each block of the proposed ADC.

**Fig. 18:**
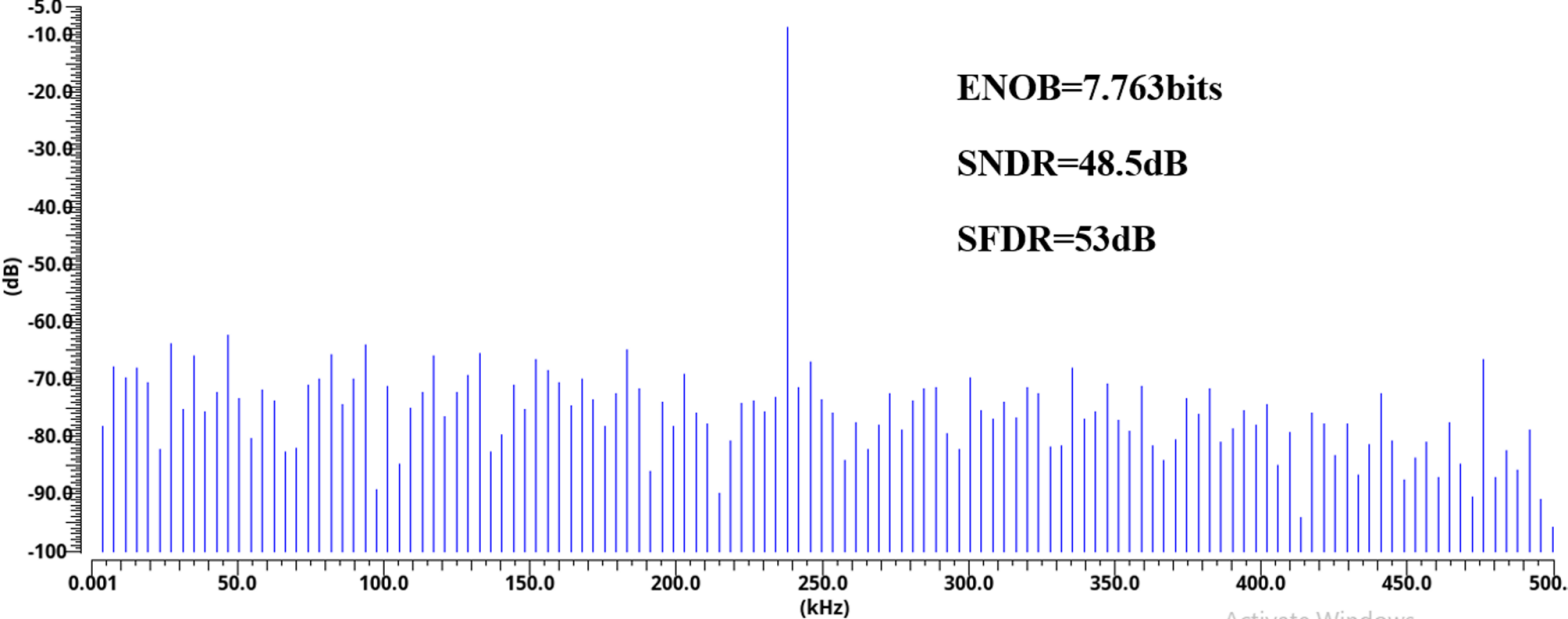
Simulated output spectra of the proposed ADC for an input frequency of 238 kHz at a sampling rate of 1 MS/s.

The performance of the proposed 8-bit low-power single-ended SAR ADC is summarized in Table 3 with the state-of-the-art SAR ADCs^4–6,12,13,17–21,36–43^ with nearly the same resolution and frequency rate to have a fair comparison. The proposed ADC exhibits spurious-free-dynamic range (SFDR) of 53 dB and signal-to-noise-and-distortion ratio (SNDR) of 48.5 dB with an effective number of bits (ENOB) is 7.763 dB. The power breakdown for each block of this ADC is shown in Fig. 17. The SAR control logic, the capacitive DAC, and the dynamic comparator consume 48%, 31%, and 20% of the total consumed power, respectively. The proposed SAR ADC achieves high energy efficiency compared with prior work demonstrated in^6,12,17,20,21,36–43^. The proposed ADC achieves 5.75 µW total post-layout power consumption. Because of including the improved S/H switch, dynamic comparator, bridge CDAC, and SAR logic control circuit, they have been optimized for power consumption and area. Figure 18 shows simulated output spectra of the proposed ADC for an input frequency of 238 kHz at a sampling rate of 1 MS/s. According to the post-simulation results that are listed in Table 3, the proposed ADC has 72.6% lower power consumption than work^20^, with the same resolution of 8-bit and a frequency rate of 1MS/s.

The well-known Walden figure of merit ($\:{FOM}_{W}$) is utilized to evaluate how efficiency power is consumed with a certain sampling rate (F_s_) and attained ENOB, Eq. (9)^10^. This FOM is useful to obtain a performance metric to compare several SAR ADCs with different resolutions and sampling rates.

9 $$\:{\varvec{F}\varvec{O}\varvec{M}}_{\varvec{W}}=\raisebox{1ex}{${\varvec{P}}_{\varvec{A}\varvec{D}\varvec{C}}$}\!\left/\:\!\raisebox{-1ex}{${2}^{\varvec{E}\varvec{N}\varvec{O}\varvec{B}}{\varvec{F}}_{\varvec{S}}$}\right.\:(\text{f}\text{J}/\text{c}\text{o}\text{n}\text{v}.-\text{s}\text{t}\text{e}\text{p})$$

**Fig. 19:**
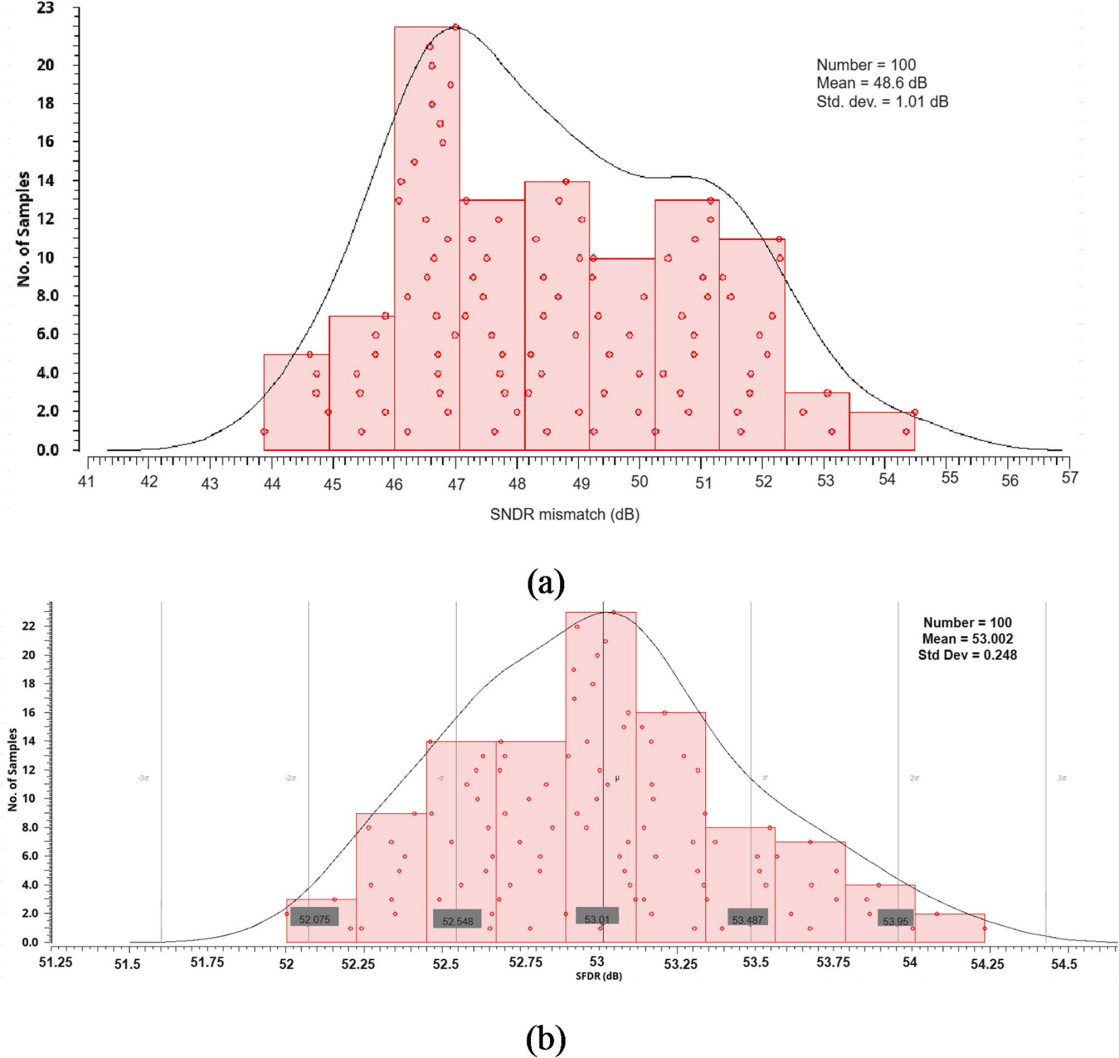
The 100-times Monte-Carlo SFDR mismatch simulation of the proposed SAR-ADC (**a**) SNDR (**b**) SFDR.

A FOM of 26.52 fJ/conversion-step is attained at 1 MS/s and a 1.2-V supply. The proposed ADC achieves the lowest FOM as compared with^4–6,13,17,20,21,36,37,41,42^, having the same number of bits. The SNDR and SFDR with 100 times Monte Carlo simulations indicate that the SNDR and SFDR normalize distribution with a 48.6 and 53 dB mean and 1.05- and 0.248-dB standard deviation, as shown in Fig. 19(a) and (b), respectively.

**Fig. 20:**
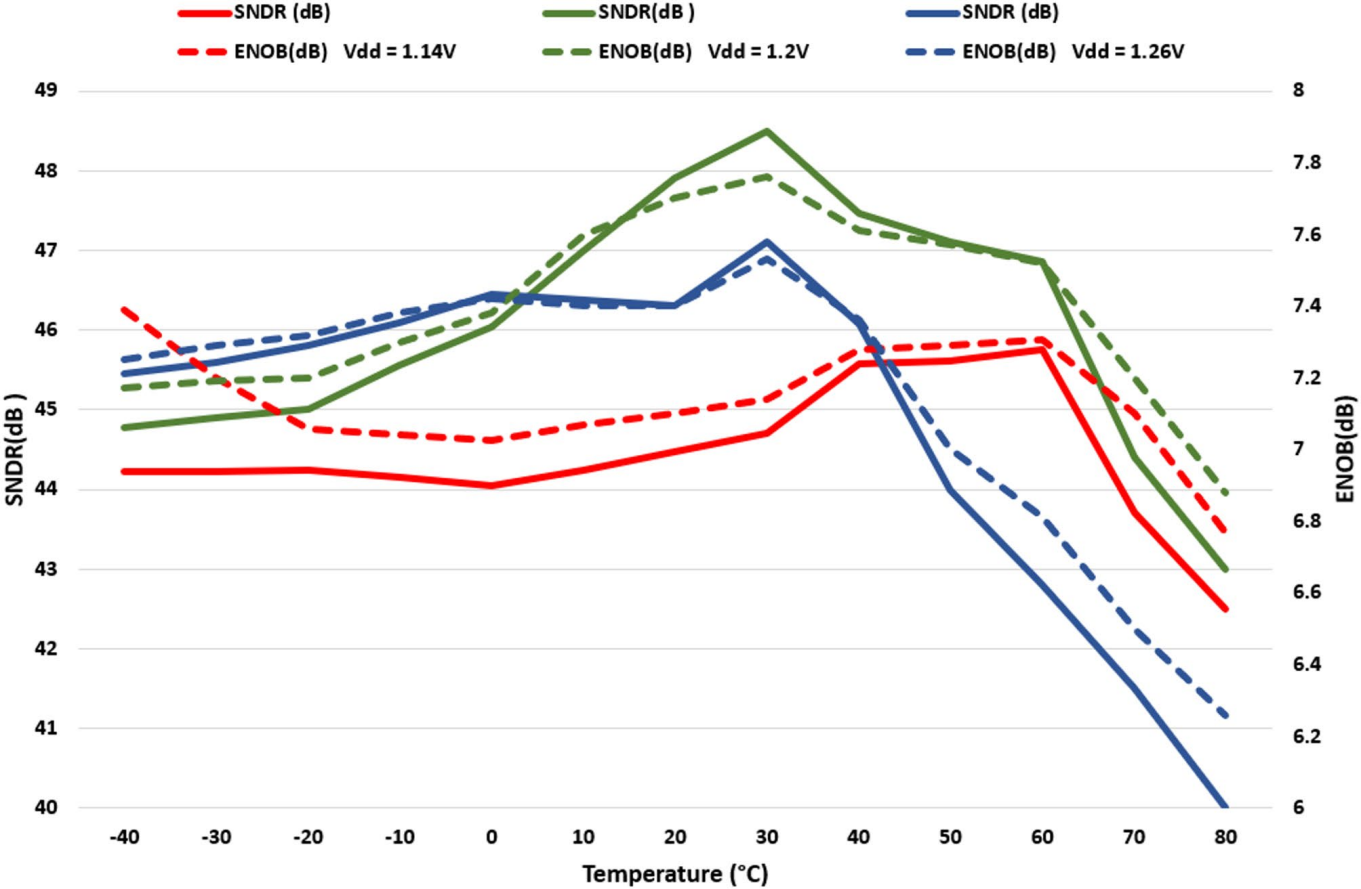
SNDR and ENOB of the proposed SAR-ADC for various temperature and voltage supply with 7.8125 kHz sinusoidal input signal.

The stability of the proposed single-SAR ADC against changes in temperature and power supply is shown in Fig. 20. It demonstrates that across the − 40 °C: 80 °C and ± 5% supply variation range, the ENOB and SNDR vary by less than 0.82 dB and 4.8 dB, respectively. Thus, it confirms the PVT stabilizing scheme’s effectiveness.

**Table 3:**
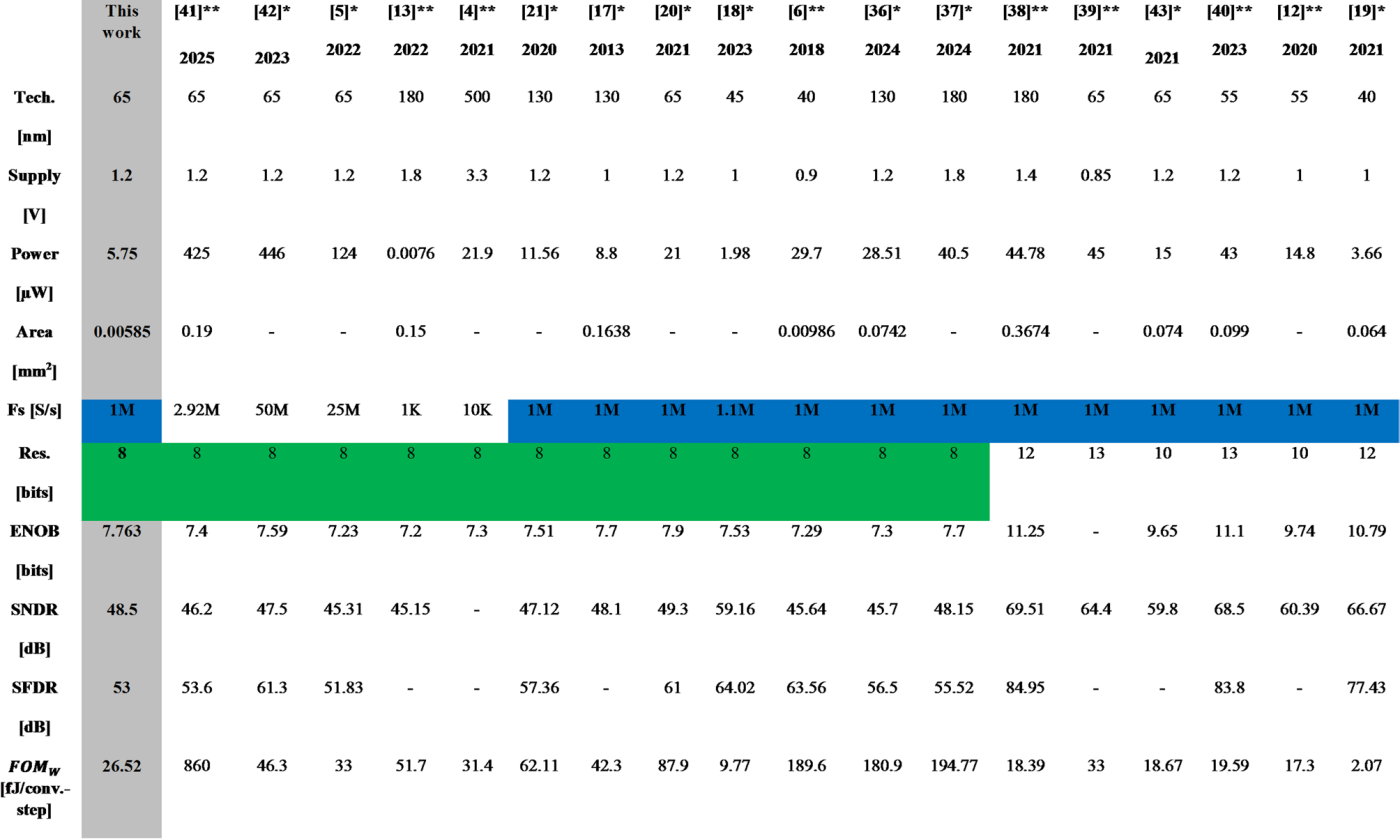
Comparison of the proposed SAR-ADC with prior SAR-ADCs.

## Conclusion

This paper presents a low-power 8-bit single-ended successive-approximation-register (SAR) analog-to-digital converter (ADC) valid for biomedical applications. The use of high-isolation S/H circuit, double-tail with extra pair PMOS transistors dynamic comparator, synchronous modified SAR logic control register based on D-FFs, and MIM modified capacitive DAC enables the ADC to boost the overall performance besides minimizing the power consumption. Implementing the proposed SAR ADC in a 65-nm CMOS process, achieves 7.763-dB ENOB, 48.5-dB SNDR, and 53-dB SFDR with sampling frequency 1MS/s. The power consumption is 5.75 µW under 1.2-V supply voltage, corresponding to a FoMs of 26.52 fJ/conversion-step. The overall active area is 120.8µm$\:\times\:$48.44 μm.

## Data Availability

The datasets used and/or analysed during the current study are available from the corresponding author on reasonable request.
